# Advancing Mammographic Screening Among Underserved Groups: A Systematic Review and Meta-Analysis of Intervention Strategies to Increase Breast Cancer Screening Uptake

**DOI:** 10.3389/phrs.2025.1607873

**Published:** 2025-04-04

**Authors:** Allegra Ferrari, Deborah Jael Herrera, Wessel Van De Veerdonk, Wendy D’haenens, Andrea Ruiz Alejos, Nigus Bililign Yimer, Sheila Orwa, Liesbet Van Bos, Sarah Talboom, Lilu Ding, Mathieu Goossens, Guido Van Hal

**Affiliations:** ^1^ Research Group Social Epidemiology and Health Policy (SEHPO), Department of Family Medicine and Population Health (FAMPOP), University of Antwerp, Antwerp, Belgium; ^2^ Department of Health Sciences (DISSAL), University of Genoa, Genoa, Italy; ^3^ Centre of Expertise–Care and Wellbeing, Campus Zandpoortvest, Thomas More University of Applied Sciences, Mechelen, Belgium; ^4^ Centre of Expertise–Sustainable Business and Digital Innovation, Campus De Ham, Thomas More University of Applied Sciences, Mechelen, Belgium; ^5^ Department of Epidemiology and Health Statistics, School of Public Health, Hangzhou Medical College, Hangzhou, China; ^6^ Centre for Cancer Detection (CvKO), Bruges, Belgium

**Keywords:** breast cancer, breast cancer screening, intervention, mammography, screening uptake

## Abstract

**Objectives:**

Breast cancer (BC) is a leading cause of cancer related disability and mortality. Despite efforts to implement mammography screening programs, uptake rates vary widely due to socioeconomic factors and accessibility challenges. To improve participation, interventions targeting barriers faced by underserved groups are crucial for promoting equitable screening and early detection.

**Methods:**

A systematic search and meta-analysis was performed to identify strategies to reduce disparities and enhance participation in BC screening, with particular attention to underserved groups, including individuals with low SES, underinsured, with immigrant background or part of ethnic minority.

**Results:**

The meta-analysis of 44 randomized studies involving 161,141 individuals (of which 14,720 belonged to underserved groups) showed that, compared to usual care, interventions regarding BC screening were effective in increasing mammography uptake [pooled OR 1.55 (95%CI 1.39–1.73)], particularly, among underserved groups [pooled OR 1.81 (95%CI 1.43–2.28)]. Overall, the most effective were educational interventions. Among underserved groups, reminders, telephonic interventions, navigation services and cultural-sensitive approaches were highly effective.

**Conclusion:**

Combining these strategies can simultaneously address multiple barriers, ensuring comprehensive support throughout the BC screening process and improved access to screening for underserved groups.

**Systematic Review Registration:**

Identifier CRD42023393352.

## Introduction

Breast cancer (BC) ranks first in terms of cancer incidence globally, with over two million cases diagnosed each year [1]. In 2019, BC was the leading cause of cancer-related disability-adjusted life years (DALYs) (20.3 million) and deaths (689,000) among females [2].

The prognosis and treatment outcomes for BC are significantly influenced by tumor characteristics and the stage at diagnosis. Evidence indicates that tumors detected via mammography screening generally have better prognostic features compared with tumors detected via other methods. Screen-detected tumors are typically at earlier stages, well-differentiated, less likely to metastasized, and exhibit lower proliferation scores [3, 4]. Even when accounting for various prognostic factors, studies indicate that participation in screening can reduce the risk of cause-specific mortality by approximately 40% [5].

Insufficient participation leads to diminished cost-effectiveness of the screening program [6]. Increasing adherence to recommended screening guidelines [7, 8] is therefore crucial for maximizing the early detection of BC and reducing mortality rates [7, 8]. However, globally, the uptake rate for BC screening varies significantly between and within regions.

In Europe, the average uptake rate of BC screening is 48.2% [9]. Despite increased efforts by the European Council since 2003, the implementation of structured, population-based mammography screening varies widely, with uptake rates ranging from 19.4% to 88.9%. This primarily depends on the laws in existence in various settings, the structure of healthcare, and the resources available [10]. In the United States (US), approximately 76.4% of women aged 50–74 reported having had a mammogram within the past 2 years in 2019. However, participation drops notably to around 40% among uninsured women, highlighting disparities influenced by state policies, demographic factors, and healthcare access [11]. In low- and middle-income countries (LMICs), BC screening programs are less widespread, resulting in lower participation rates and higher mortality. Contributing factors to these disparities include limited access to screening facilities, lack of awareness about early detection, and socioeconomic barriers [12–14].

Underserved groups, including individuals from vulnerable or marginalized communities such as racial and ethnic minorities, immigrants, low-income individuals, and those with limited health literacy, often face significant challenges to access preventive health services [15–17]. Addressing their multifaceted challenges requires targeted interventions that extend beyond mere accessibility. Possible strategies include enhancing awareness about the importance of early detection, improving affordability of screening services through subsidies or insurance coverage, and ensuring linguistic and culturally sensitive healthcare practices [18].

Implementing evidence-based interventions tailored to the specific needs of diverse communities can potentially help healthcare systems foster trust, reduce disparities, and ultimately improve BC screening rates and health outcomes. However, the extent to which tailored interventions increase mammography uptake across varying strategies and populations remains unclear.

### Objectives

• Our primary objective was to systematically analyze existing literature on the interventions to increase BC screening (participation in BC screening programs, mammography uptake) and, where possible, to perform meta-analyses of the effectiveness of these interventions, by type of intervention implemented, underserved status of the target population, and other relevant contextual variables.
• Secondarily, our objective was to report on additional effects that interventions to increase BC screening might have on other relevant outcomes including the performance of breast self-examination (BSE) and clinical breast examination (CBE).

## Methods

### Systematic Review Protocol

The protocol of this systematic review was registered in the International Prospective Register of Systematic Reviews (PROSPERO registration number: CRD42023393352). The study adhered to the PRISMA (Preferred Reporting Items for Systematic Reviews and Meta-Analyses) guidelines for transparent and comprehensive reporting [19].

### Eligibility Criteria

In this review, the Population, Intervention, Comparator, Outcome, and Study design (PICOS) system was used to develop the literature search strategy [20].

• Population: Apparently healthy or asymptomatic females in the target age groups (which may differ in different countries) for BC screening. Both the general population and underserved groups are eligible for inclusion. Underserved groups are identified by characteristics such as low-SES, underinsurance, immigrant background, ethnic minority status, or cultural/religious barriers, present in the majority (>50%) of the study population.
• Intervention: Interventions to increase uptake of BC screening are structured efforts designed to increase the uptake and adherence to BC screening programs, particularly mammography. These can include, but are not limited to: educational interventions, patient navigation programs, reminder systems, policies and insurance interventions, and cultural and linguistic adaptations.
• Comparator: Standard care, no intervention, or alternative interventions aimed at increasing BC screening rates. The closest to standard care is considered when a study has more than one comparison group.
• Outcome: Effectiveness of interventions in increasing BC screening uptake (participation in BC screening programs, mammography use).
• Study design: Quasi-experimental study design/quasi-randomized control designs, Randomized control trials (RCTs), Cluster randomized control trials and non-randomized control trials, controlled before-after studies (CBAs)

Exclusion criteria included non-English language studies, studies that did not investigate outcomes related to mammography uptake, and studies focused on breast cancer survivors or other individuals not considered healthy. Research papers that concentrate on treatment conditions, rehabilitation, non-intervention studies, biomedical or treatment research, pharmaceutical research, descriptive research, and those lacking valid outcome measures were also excluded.

### Search Strategy and Data Sources

The following databases were searched: Cochrane Central Register of Controlled Trials, Medline (via Ovid), Global Health (via Ovid), Biological Abstracts (via Ovid), Scopus, Web of Science citation indexing, Google Scholar.

This review aimed to fill the temporal gap left after Agide et al.’s groundwork that included 22 studies published between 2004 and 2016 [21]. These reports were assessed for eligibility according to our selected inclusion criteria. Ultimately, 20 of these studies were included in our analysis. In order to address the temporal gap left, our investigation was extended to include all eligible papers published between 2017 and 2023. The full search string is available in Supplementary Table S1.

### Study Selection

**FIGURE 1:**
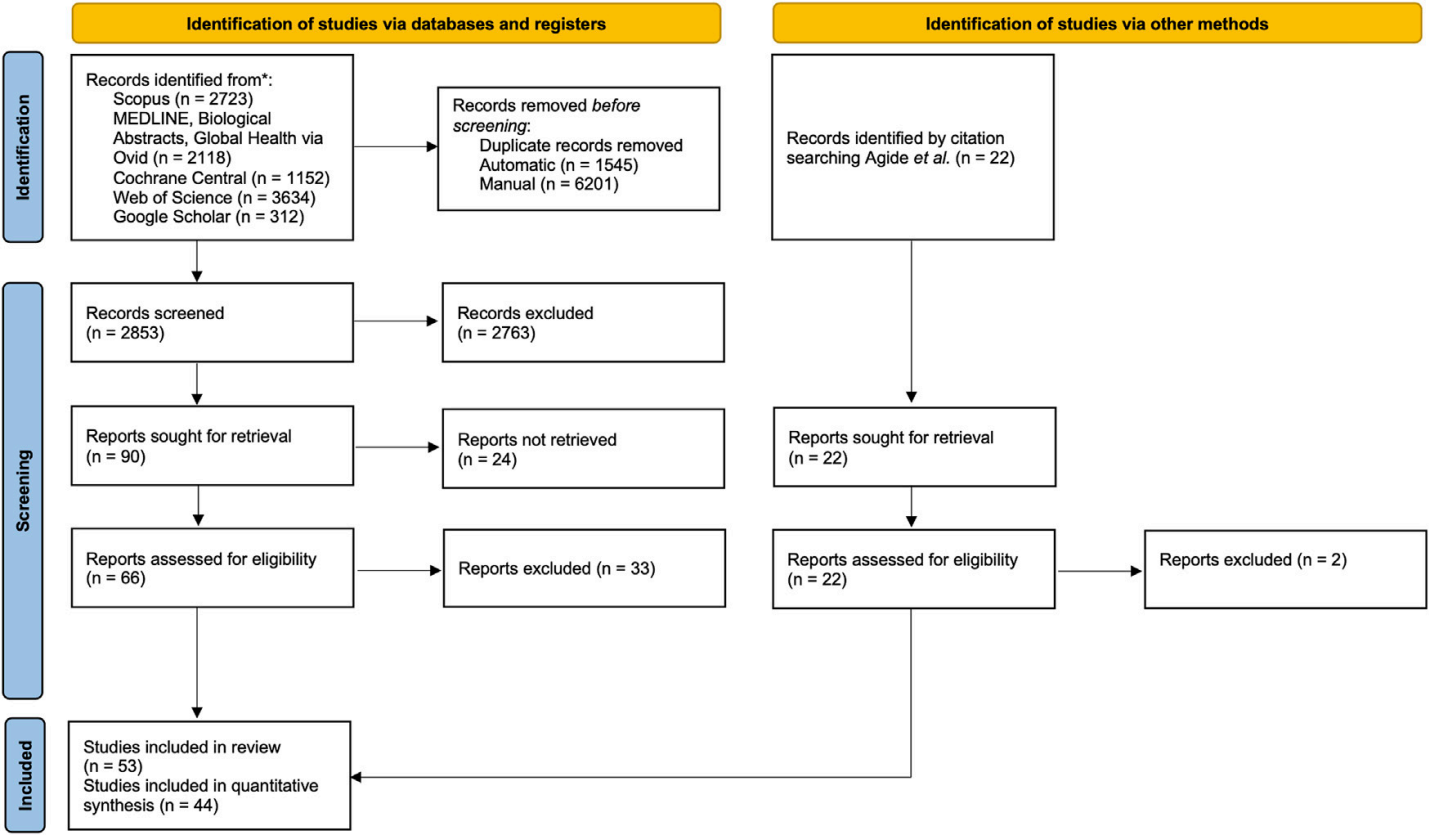
PRISMA flow chart of the screening process. Advancing Mammographic Screening Among Underserved Groups: A Systematic Review and Meta-Analysis of Intervention Strategies to Increase Breast Cancer Screening Uptake, Belgium, 2025.

Articles retrieved from the electronic databases were exported directly as MS Excel files and imported into Rayyan software for de-duplication and screening [22]. At least two independent reviewers screened the abstract (AF, ARA) and full-text articles (AF, ARA, SO, NY, DJH). The review process is presented in the PRISMA Flow chart (Figure 1).

### Data Extraction

Prior to the actual data extraction two independent reviewers (AF, SO) developed the data extraction form. This was adapted from the *Cochrane Effective Practice and Organisation of Care (EPOC)* guidelines [23]. The form underwent pilot testing, following which all relevant data were extracted in duplicate. The completion of the data extraction forms involved at least two independent review authors (AF, ARA, SO, NY, DJH). Any conflicts that arose during the review process were resolved through consensus. A third author (GVH) was consulted for arbitration when consensus was not reached.

### Risk of Bias Assessment

To appraise the quality of the randomized studies included, the Risk of Bias tool (RoB-2) for randomized controlled trials (RoB-2), developed by the Cochrane Collaboration, was used [24]. To appraise non-randomized studies, the Risk of Bias in Non-randomized Studies of Interventions (ROBINS-I) tool was used [25]. The risk of bias for each eligible study was assessed by at least two independent review authors (AF, DJH, WD). Judgments were categorized as “low risk,” “some concerns,” “high risk” for each study appraised using RoB-2 tool, and “low risk,” “moderate risk,” “serious risk,” “critical risk” for each study appraised with the ROBINS-I tool. Judgments were categorized as “No information” when details about the methods were insufficient, lacking, or not applicable. All conflicts were resolved by consensus, and a fourth author (GVH) was invited to arbitrate where necessary.

### Analysis

For studies examining categorical outcomes, we extracted proportions, percentages, and unadjusted odds ratios (ORs) along with 95% confidence intervals (CIs). When available, adjusted odds ratios (aORs) and their 95% CIs from multivariable analyses were also extracted.

A meta-analysis of randomized studies was conducted using MetaXL Version 5.3 (EpiGear International 2016) [26]. These findings were visualized using forest plots.

To account for study-level variability in the outcomes, random-effects meta-analysis was used [27]. All estimates were pooled and presented as OR (95%CI) and p-values.

#### Sensitivity Analysis

Sensitivity analyses were conducted where possible and for each subgroup to assess the robustness of the results. These analyses specifically accounted for the potential influence of studies with a high risk of bias, as assessed using the ROB-2 tool.

#### Subgroup Analyses

Subgroup analysis were performed for both population type (overall population, underserved) and intervention type (educational interventions, telephonic interventions, navigation services, invitation letters, reminders, linguistically adapted interventions, culturally sensitive interventions, digital-based educational interventions, smartphone-based interventions, decision aids, printed materials).

Where possible [28, 29], studies presenting results for multiple interventions or populations were included separately in the analysis (labelled in letters) and were therefore presented in distinct forest plots. If a study used more than one comparator, the comparator closest to standard care was chosen. For studies with different follow-up periods, we considered the longest follow-up. Detailed explanations of the studies included in the meta-analysis, as well as a comprehensive list of interventions and comparators, can be found in the Supplementary Figures S1–S13; Supplementary Table S5).

#### Assessment of Heterogeneity and Publication Bias

The amount of heterogeneity was quantified and evaluated critically by means of I^2^ (with CI) and Cochran’s Q (with p-value) [27]. Additionally, exploratory subgroup analyses were conducted for variables such as follow-up time, age of the population, and geographical area; however, these did not reveal significant trends and were therefore not included in this report. A random-effects model was applied to account for unexplained heterogeneity.

To reduce the risk of publication bias and identify as much relevant evidence as possible, the initial electronic search strategy was supplemented by citation mining and reference checking from eligible included studies. Furthermore, funnel plots were realized when applicable (≥10 studies per outcome/subgroup). These are presented in Supplementary Figures S1–S13.

### Certainty of Evidence

To assess how much confidence to place in the findings on the effectiveness of the interventions, the ‘Grading of Recommendations, Assessment, Development, and Evaluation’ (GRADE) approach was used by the review authors [30].

## Results

In total, 9,939 articles were retrieved. After deduplication, 2,853 articles, were screened based on titles and abstracts, and 88 articles underwent full-text screening. A total of 53 articles were included in the review, and 44 studies were fit for quantitative synthesis.

Studies presenting results for multiple interventions, comparators or target groups were analyzed separately (labelled in letters) and are, therefore, presented in multiple forest plots (Table 1; Supplementary Table S3, S4; Supplementary Figures S1–S13).

**TABLE 1:** Characteristics of included studies on interventions to increase mammography uptake. Advancing Mammographic Screening Among Underserved Groups: A Systematic Review and Meta-Analysis of Intervention Strategies to Increase Breast Cancer Screening Uptake, Belgium, 2025.

Author/year	Country	Design	Sample size	Target ages	Follow-up (months)	Intervention type	Conceptual framework	Underserved group	Absolute effect		Relative effect	p value
									Intervention group	Control group		
*Randomized studies*												
Abood et al. [31]	US	QES	1,104	50–64	6	Telephonic intervention	Loss-framed approach	Low-SES	31/112 (27.7%)	157/992 (15.8%)	OR 1.91 (95%CI 1.20–3.05)	0.006
Alameer et al. [32]	Saudi Arabia	QES	68	20+	3	Educational intervention	Health belief model		27/36 (75.0%)	11/32 (34.4%)	OR 5.73 (95%CI 2.0–16.35)	0.001
Alizadeh-Sabeg et al. [33]	Iran	CRT	120	40+	2	Educational intervention	Motivational interviewing, Trans-theoretical model		16/60 (26.7%)	0/60 (0.0%)	OR 44.86 (95%CI 2.62–767.93)	0.009
Allgood et al. [34]	UK	RCT	22,828	50–70	6	Invitation letter, Reminder			8,511/11,383 (74.8%)	8,254/11,445 (72.1%)	OR 1.14 (95%CI 1.08–1.22)	<0.001
Allgood et al. [35]	UK	RCT	26,054	50–70	6	Invitation letter, Reminder			3,054/12,807 (24.0%)	1784/13,247 (13.0%)	OR 1.77 (95%CI 1.67–1.88)	<0.001
Beauchamp et al. [36] (A)	Australia	RCT	1,032	50–75	0.5	Invitation letter Linguistically adapted intervention, Reminder		Immigrant	214/572 (37.4%)	179/460 (38.9%)	OR 0.94 (95%CI 0.73–1.21)	0.622
Beauchamp et al. [36] (B)	Australia	RCT	195	50–75	0.5	Telephonic intervention, Navigation, Linguistically adapted intervention, Reminder		Immigrant	61/95 (64.2%)	6/100 (6.0%)	OR 28.10 (11.14–70.90)	<0.0001
Bourmaud et al. [37]	France	RCT	15,844	50–74	12	Decision aid, Printed materials (card, brochure, leaflet, flyer)			3,174/7,885 (40.2%)	3,353/7,959 (42.1%)	OR 0.86 (95%CI 0.79–0.94)	0.001
Bowen et al. [38]	US	RCT	672	40–74	12	Decision aid	Self-regulation model of health behavior		274/334 (82.0%)	237/338 (70.0%)	OR 1.94 (1.35–2.79)	0.001
Chambers et al. [39]	Scotland	RCT	856	50–70	3	Telephonic intervention, Navigation, Reminder		Low-SES	87/639 (13.6%)	15/217 (6.9%)	OR 2.12 (95%CI 1.20–3.75)	0.001
Champion et al. [40] (A)	US	RCT	207	51–75	6	Digital-based educational intervention (web, DVD, other platforms.)	Health Belief Model and Transtheoretical Model		72/104 (69.2%)	29/103 (28.1%)	OR 0.60 (95%CI 0.37–0.97)	0.038
Champion et al. [40] (B)	US	RCT	316	51–75	6	Telephonic intervention	Health Belief Model and Transtheoretical Model		97/213 (45.5%)	48/103 (46.6%)	OR 0.95 (95%CI 0.59–1.53)	0.859
Champion et al. [40] (V-A)	US	RCT	486	51–75	6	Digital-based educational intervention (web, DVD, other platforms)	Health Belief Model and Transtheoretical Model	Low-SES	134/325 (41.2%)	57/161 (35.4%)	OR 1.28 (95%CI 0.86–1.89)	0.216
Champion et al. [40] (V-B)	US	RCT	491	51–75	6	Telephonic intervention	Health Belief Model and Transtheoretical Model	Low-SES	113/329 (34.5%)	58/162 (35.8%)	OR 1.21 (95%CI 0.82–1.79)	0.323
Champion et al. [40] (A)	US	RCT	191	51–75	1	Decision aid	Theory of Planned Behavior, Health Belief Model, and Transtheoretical Model		39/125 (31.2%)	16/66 (24.2%)	OR 1.40 (95%CI 1.21–2.34)	0.038
Champion et al. [40] (B)	US	RCT	178	51–75	1	Telephonic intervention	Theory of Planned Behavior, Health Belief Model, and Transtheoretical Model		41/133 (30.1%)	16/65 (24.6%)	OR 1.38 (95%CI 1.20–2.37)	0.859
Champion et al. [41]	US	RCT	245	50–74	12	Digital-based educational intervention (web, DVD, other platforms.), Navigation			87/162 (54.0%)	25/83 (30.0%)	OR 5.11 (95%CI 2.57–10.60)	<0.001
Chan et al. [42]	Canada	RCT	5,498	51–73	6	Invitation letter, Reminder			974/2,749 (34.4%)	660/2,749 (24.0%)	RR 1.41 (95%CI 1.30–1.54)	<0.0001
Coronado et al. [43]	US	RCT	928	42–74	12	Educational intervention, Culturally sensitive intervention		Ethnic minority	86/439 (19.6%)	48/489 (11.0%)	OR 2.24 (95%CI 1.53–3.27)	<0.0001
Elder et al. [44]	US	CRT	436	40–65	24	Educational intervention, Culturally sensitive intervention, Linguistically adapted intervention		Ethnic minority, Immigrant, Low-SES, Low-literacy	134/219 (61.0%)	91/217 (42.0%)	OR 4.64 (95%CI 2.00–10.75)	0.0004
Elliot et al. [45]	US	CRT	2003	21–74	7.5	Decision aid			NA	NA	OR 0.91 (95%CI 0.66–1.21)	0.46
Fernandez et al. [46]	US	RCT	343	40+	6	Telephonic intervention, Navigation	Social Cognitive Theory		87/211 (41.2%)	46/132 (34.8%)	OR 1.53 (95%CI 0.91–3.59)	0.11
Freund et al. [47] (A)	Israel	RCT	598	40–60	3	Telephonic intervention, Culturally sensitive intervention	Culture-Based Health Belief		70/389 (18.0%)	15/209 (16.6%)	OR 1.1 (95%CI 11–1.8)	0.0005
Freund et al. [47] (B)	Israel	RCT	331	40–60	3	Telephonic intervention, Culturally sensitive intervention	Culture-Based Health Belief	Ethnic minority	42/241 (17.42%)	5/90 (5.55%)	OR 3.59 (95%CI 1.37–9.38)	0.009
Goel et al. [48]	US	RCT	194	40+	12	Digital-based educational intervention (web, DVD, other platforms), Navigation, Linguistically adapted intervention	Social Cognitive Theory	Ethnic minority	32/97 (33.0%)	13/97 (13.0%)	OR 5.21 (95%CI 1.6–17.1)	0.007
Goldzahl et al. [49]	France	RCT	21,195	50–74	12	Invitation letter	Social norms framework		7,451/15,918 (46.8%)	2,510/5,277 (48.6%)	OR 0.97 (95%CI 0.91–1.03)	0.340
Goossens et al. [50] (A)	Belgium	QES	3,011	50–74	3	Invitation letter			224/1,501 (14.9%)	99/1,510 (6.6%)	RR 2.3 (95%CI 1.80–2.88)	<0.001
Goossens et al. [50] (B)	Belgium	QES	820	50–74	3	Invitation letter			41/410 (10.0%)	23/410 (5.6%)	RR 1.8 (95%CI 1.07–2.97)	0.026
Goossens et al. [50] (C)	Belgium	QES	967	50–74	3	Invitation letter			166/483 (34.4%)	90/484 (18.6%)	RR 1.8 (95%CI 1.43–2.39)	<0.001
Hajian et al. [51]	Iran	RCT	100	20–60	3	Educational intervention	Health belief model		18/50 (36.0%)	15/50 (30.0%)	OR 1.31 (95%CI 0.57–3.03)	0.524
Holt et al. [52]	US	CRT	382	40–75	24	Educational intervention, Culturally sensitive intervention		Ethnic minority	188/191 (98.6%)	186/191 (97.5%)	OR 0.96 (95%CI 0.47–1.96)	0.47
Kim et al. [53]	South Korea	QES	121	30–64	6	Educational intervention, Navigation		Immigrant	19/61 (13.1%)	0/60 (0.0%)	OR 55.51 (95%CI 3.26–944.96)	0.005
Kiran et al. [54]	Canada	RCT	1,175	50–74	6	Telephonic intervention, Reminder			164/591 (27.8%)	138/584 (23.6%)	OR 1.24 (95%CI 0.95–1.61)	0.106
Lee et al. [55]	US	RCT	55	40–50	2	Digital-based educational intervention (web, DVD, other platforms), Family member intervention, Linguistically adapted intervention		Immigrant	5/23 (21.7%)	4/32 (12.5%)	OR 1.94 (95%CI 0.46–8.22)	0.366
Lee et al. [56]	US	RCT	120	40–79	6	Smartphone-based interventions (apps/SMS/social media campaigns), Navigation	Health belief model	Ethnic minority	45/60 (75.0%)	18/60 (30.0%)	OR 1.60 (95%CI 1.10–1.70)	<0.001
Lin et al. [83]	Taiwan	QES	108	45+	3	Telephonic intervention, Navigation			21/48 (43.7%)	8/60 (13.3%)	OR 5.06 (95%CI 2.04–13.57)	0.001
Lin et al. [83]	Taiwan	QES	106	45+	3	Invitation letter, Reminder			16/46 (34.8%)	8/60 (13.3%)	OR 3.47 (95%CI 1.36–9.46)	0.011
Luckmann et al. [57]	US	RCT	30,160	40–84	48	Invitation letter, Telephonic intervention, Navigation, Reminder			16,460/20,097 (81.9%)	8,131/10,063 (80.8%)	OR 1.07 (95%CI 1.01- 1.14)	0.020
Margulies et al. [58]	US	RCT	49	40–76	0.5	Health consultation, Navigation			19/25 (76.0%)	10/24 (42.0%)	Or 4.43 (95%CI 1.30–15.09)	0.017
Marshall et al. [59]	US	RCT	1,358	65+	24	Navigation		Ethnic minority	595/638 (93.3%)	630/720 (87.5%)	OR 2.26 (95%CI 1.59–3.22)	<0.001
Mirmoammadi et al. [60]	Iran	CRT	150	40+	3	Educational intervention	Health Belief Model, GATHER consultancy technique		37/75 (49.3%)	15/75 (20.0%)	OR 3.89 (95%CI 1.89–8.03)	0.240
Montero-Moraga et al. [61]	Spain	CRT	11,087	50–69	6	Decision aid			1964/5,393 (36.4%)	2,135/5,694 (37.5%)	OR 0.95 (0.88–1.03)	<0.0001
Nanda et al. [62]	US	RCT	1,277	50–65	3	Smartphone-based interventions (apps/SMS/social media campaigns), Navigation, Reminder			85/843 (10.1%)	23/434 (6.2%)	OR 2.00 (95%CI 1.24–3.22)	0.004
Nanda et al. [63]	US	RCT	880	50–65	6	Telephonic intervention, Navigation			100/438 (23.0%)	53/442 (12.0%)	OR 2.17 (95%CI 1.51–3.12)	<0.0001
Nguyen et al. [64]	US	RCT	1,089	40+	24	Educational intervention, Navigation, Reminder		Ethnic minority	446/543 (82.1%)	413/546 (75.6%)	OR 3.14 (95%CI 1.98–5.01)	<0.001
Pérez-Lacasta et al. [65]	Soain	CRT	400	49–50	3	Decision aid			128/203 (63.1%)	129/197 (65.5%)	OR 2.00 (95%CI 1.24–3.22)	0.004
Roberto et al. [66]	Italy	RCT	1,001	45+	NA	Decision aid	International Patient Decision Aid Standards Collaboration. Nudging-like approach		376/472 (84.1%)	416/529 (83.0%)	OR 1.06 (95%CI 0.78–1.44)	0.691
Schapira et al. [67]	US	RCT	113	39–48	12	Decision aid	Theoretical framework of SDM and Exemplification theory	Ethnic minority	13/54 (24.1%)	13/59 (22.0%)	OR 1.12 (95%CI 0.46–2.69)	0.797
Sinicrope et al. [68]	US	RCT	25	40+	3	Educational intervention, Linguistically adapted intervention		Ethnic minority	B7/13 (54.0%)	4/12 (34.1%)	OR 2.33 (95%CI 0.46–11.80)	0.306
Slater et al. [69]	US	RCT	4,793	65–84	12	Printed materials (card, brochure, leaflet, flyer), Economic support	Transtheoretical model	Uninsured or underinsured	NA	NA	OR 1.36 (95%CI 1.18–1.56)	NA
Taymoori et al. [70] (A)	Iran	RCT	93	50+	6	Educational intervention	Health belief model		37/63 (59.0%)	7/30 (23.3%)	OR 4.88 (95%CI 1.83–13.00)	0.001
Taymoori et al. [70] (B)	Iran	RCT	91	50+	6	Educational intervention	Theory of planned behavior		39/60 (65.0%)	7/31 (22.6%)	OR 6.36 (95%CI 2.36–17.22)	0.0003
Tuzcu et al. [71]	Türkiye	QES	190	20+	6	Digital-based educational intervention (web, DVD, other platforms), Invitation letter	Health Belief Model and Health Promotion Model	Immigrant	20/91 (22.0%)	9/99 (9.1%)	OR 2.82 (95%CI 1.21–6.56)	0.016
Wyatt et al. [72]	US	RCT	421	40–75	4	Educational intervention, Navigation, Culturally sensitive intervention, Linguistically adapted intervention	Social marketing theory	Ethnic minority, Possible religious barriers	101/210 (49.0%)	91/211 (44.6%)	OR 1.22 (95%CI 0.83–1.79)	0.306
*Non-randomized studies*												
Brown et al. [73]	US	CBA	226	50–74	6	Health consultation, Navigation, Economic support/Voucher		Ethnic minority, Uninsured or underinsured	37/68 (54.0%)	74/158 (47.0%)	OR 1.35 (95% CI 0.76–2.40)	0.297
Cohen et al. [74]	Israel	QES	40	40–65	6	Telephonic intervention, Culturally sensitive intervention	Transtheoretical model, Health belief model, Cultural competence approach	Ethnic minority, Possible cultural or religious barriers	10/26 (38.5%)	3/14 (21.4%)	OR 2.29 (95%CI 0.51–10.28)	0.279
Falk et al. [75] (A)	US	QES	3,082	40–74	6	Navigation		Ethnic minority	1,358/1828 (74.3%)	356/1,254 (28.4%)	OR 6.06 (95%CI 4.87–7.53)	<0.001
Falk et al. [75] (B)	US	QES	3,114	40–74	6	Educational intervention, Navigation		Ethnic minority	1,170/1860 (62.9%)	356/1,254 (28.4%)	OR 3.33 (95%CI 2.77–4.02)	<0.001
Fleming et al. [76]	Ireland	QES	204,196	50–64	12	Invitation letter			75,375/102,393 (74.0%)	77,702/101,803 (76.0%)	OR 0.86 (95%CI 0.85–0.88)	<0.0001
Kizilkaya et al. [77]	Türkiye	CBA	200	40+	6	Educational intervention, Navigation		Immigrant, low literacy	100/100 (100%)	0/100 (0.0%)	—	—
Molina et al. [78]	US	QES	126	52–74	6	Educational intervention, Navigation, Culturally sensitive intervention	Social cognitive theory, Volunteerism, Cognitive dissonance theory	Ethnic minority	55/64 (72.0%)	33/62 (48.0%)	OR 3.11 (95%CI 1.40–6.91)	0.005
Mosavel et al. [79]	US	QES	19	40+	3	Educational intervention, Family member intervention	Elaboration Likelihood Model, Theory of Planned Behavior	Ethnic minority, Uninsured or underinsured	5/12 (42.0%)	2/7 (28.6%)	OR 6.25 (95%CI 0.61–63.54)	0.121
Ramirez et al. [80] (A)	US	CBA	4,342	41+	5	Smartphone-based interventions (apps/SMS/social media campaigns)			185/NA	173/NA	OR 1.72 (95%CI 0.92–3.22)	0.087
Ramirez et al. [80] (B)	US	CBA		41+	5	Smartphone-based interventions (apps/SMS/social media campaigns), Navigation			206/NA	187/NA	OR 3.08 (95%CI 1.47–6.46)	0.003
Ramirez et al. [80] (C)	US	CBA		41+	5	Smartphone-based interventions (apps/SMS/social media campaigns), Navigation, Educational intervention			175/NA	167/NA	OR 2.33 (95%CI 1.29–4.23 (	0.005
Ramirez et al. [80] (V-A)	US	CBA		41+	5	Smartphone-based interventions (apps/SMS/social media campaigns)		Uninsured or underinsured	283/NA	244/NA	OR 1.83 (95%CI 0.88–3.80)	0.106
Ramirez et al. [80] (V-B)	US	CBA		41+	5	Smartphone-based interventions (apps/SMS/social media campaigns), Navigation		Uninsured or underinsured	283/NA	241/NA	OR 2.04 (95%CI 1.03–4.05)	0.042
Ramirez et al. [80] (V-C)	US	CBA		41+	5	Smartphone-based interventions (apps/SMS/social media campaigns), Navigation, Educational intervention		Uninsured or underinsured	287/NA	262/NA	OR 2.57 (95%CI 1.42–4.66)	0.002
Savicka et al. [81]	Latvia	CBA	1,064	50–69	6	Telephonic intervention, Media campaign	Nudge approach		226/674 (33.53%)	212/390 (54.4%)	OR 0.42 (95%CI 0.33–0.55)	<0.0001

Abbreviations: QES, Quasi-Experimental Study Design; RCT, Randomized Controlled Trial; CRT, Cluster Randomization Trial; CBA, Controlled before-after study; OR, Odds ratio; CI, Confidence Interval.

Underserved status of the target group was considered if the majority (>50%) presented one of the following characteristics: low-SES, being underinsured or uninsured, having an immigrant background, belonging to an ethnic minority, or facing possible cultural or religious barriers.

### Study Characteristics

Of the 53 studies, 44 employed randomized designs [31–72, 82, 83] while 9 used non-randomized designs [73–81].

Several studies were conducted in the United States (n = 28) [31, 38, 40, 41, 43–46, 48, 52, 55–59, 62, 64, 67–69, 72, 73, 75, 78–80, 82], Canada [42, 54], and Australia [36], as well as in Europe, including Italy [66], Spain [61, 65], France [37, 49], Belgium [50], Latvia [81], UK [34, 35, 39], Ireland [76]. Furthermore, studies were conducted in the Middle East including Iran [33, 51, 60, 70], Türkiye [71, 77], Saudi Arabia [32], and Israel [74], and East Asia, including South Korea [53] and Taiwan [83].

Of the 54 included studies, 26 focused on underserved groups, including ethnic minorities (n = 15) [43, 44, 48, 52, 56, 59, 64, 67, 68, 72–75, 78, 79], immigrant communities (n = 6) [36, 44, 53, 56, 71, 77], with low-literacy or other cultural or religious barriers (n = 5) [44, 47, 72, 74, 77], with low-SES (n = 4) [31, 39, 44, 82], uninsured or underinsured (n = 4) [69, 73, 79, 80].

While most studies focused on populations falling in the target age groups commonly identified for BC screening of 45–74 years [84], 24 studies also included younger individuals (from 18 years on) [32, 33, 38, 43, 45–48, 51–53, 56, 57, 60, 64, 67, 68, 71, 72, 74, 75, 77, 79, 80] and 3 studies included older individuals (up to 84 years) [56, 57, 69].

Most studies followed-up participants for a period comprised between 1 week and 3 months (n =19) [32, 33, 36, 39, 40, 47, 48, 50, 51, 56, 58, 60, 62, 65–68, 79, 83] or between 4 and 6 months [34, 35, 42, 53, 54, 61, 62, 71–75, 77, 81, 82] (n =15). However, there were also studies with a follow-up period comprised between 7 and 12 months [37, 38, 41, 43, 45, 48, 49, 66, 67, 69, 76] or of over a year [44, 52, 57, 59, 64].

The complete list of study characteristics is shown in Table 1.

### Types of BC Screening Intervention

In total, 14 types of interventions aimed at increasing BC screening uptake were identified.

In particular, 25 studies employed navigation services [36, 39, 41, 46, 48, 53, 56–59, 62–64, 69, 72, 73, 75, 77, 78, 80, 83], 16 studies employed educational interventions [32, 43, 44, 51–53, 60, 64, 68, 70, 72, 75, 77–80], 13 employed telephonic interventions [31, 36, 39, 40, 46, 47, 54, 57, 63, 74, 81–83], 12 employed reminders [34, 36, 39, 40, 54, 57, 62, 64, 71, 73, 80, 83], 10 employed invitation letters [34–36, 42, 49, 50, 57, 71, 76, 83], 8 employed decision aids [37, 38, 40, 45, 61, 65–67], 7 employed linguistically adapted strategies (e. g., using materials translated into different languages, using culturally appropriate language or employing communication strategies effective for speakers of a particular language or dialect) [36, 44, 48, 55, 68, 72, 73], 7 employed culturally sensitive approaches (e. g., tailored to align with the values and religious beliefs and practices of a specific community or group) [43, 44, 47, 52, 72, 74, 78], 5 employed digital-based interventions (e. g., web, DVD, other platforms) [41, 48, 55, 71, 82], 4 employed smartphone based-interventions (apps/SMS/social media campaigns) [56, 63, 80, 81], 3 employed printed materials (card, brochure, leaflet, flyer) [37, 57, 69], 2 employed health consultations with physicians [58, 73], 2 gave economic support or vouchers for testing [69, 73] and 2 asked for the mediation of a family member [56, 79].

### Frameworks and Models Utilized in BC Screening Interventions

Of the 23 studies describing interventions based on a specific conceptual framework or model, 9 employed the Health Belief Model (HBM) [32, 40, 51, 56, 60, 70, 71, 74, 82]. The following most common were the Transtheoretical Model (TTM) (n = 5) [33, 40, 69, 74, 82], the Theory of Planned Behavior (TBP) (n = 3) [40, 70, 79] and the Social Cognitive Theory (SCT) (n = 3) [46, 48, 78]. Interventions were also based on the Nudge approach [66, 81], Theoretical frameworks of decisions aids [66, 67], Volunteerism [78], the Cognitive Dissonance Theory [78], Culture-Based Health Beliefs [47], the Social marketing theory [72], the GATHER Consultancy Technique [60], Elaboration Likelihood Model [79], the Exemplification theory [67], a Cultural Competence approach [74], the Health Promotion Model [71], the Social norms theory [49], the Self-regulation model of Health Behavior [38], Motivational interviewing [32] and a Loss-framed approach [31].

### Risk of Bias Assessment

**FIGURE 2:**
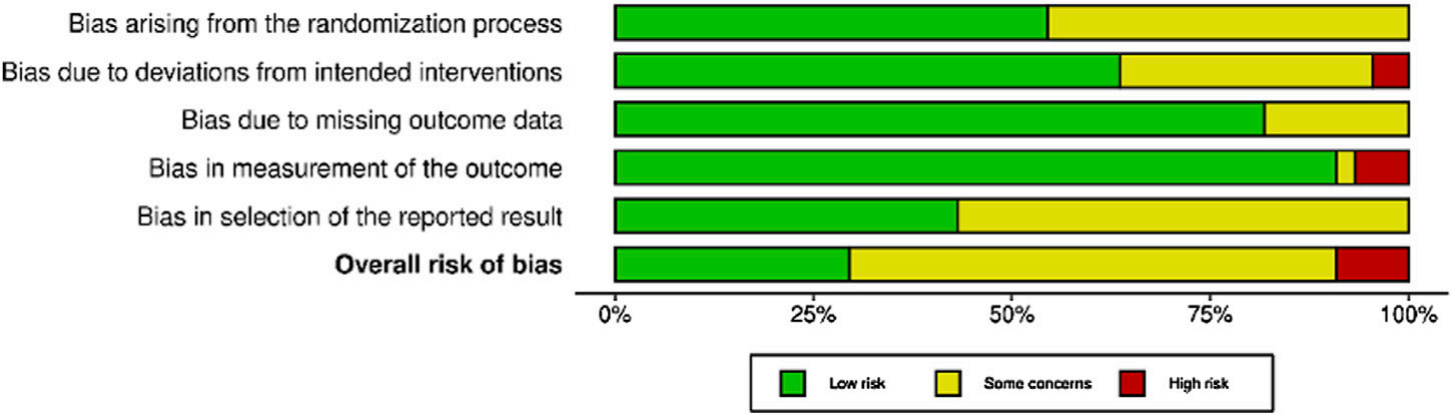
Risk of bias in randomized studies of interventions (RoB-2). Advancing Mammographic Screening Among Underserved Groups: A Systematic Review and Meta-Analysis of Intervention Strategies to Increase Breast Cancer Screening Uptake, Belgium, 2025.

Among randomized studies appraised with the ROB-2 tool (Figure 2), 12 were judged to be at low risk of bias [34–36, 40, 45, 46, 52, 62, 63, 65, 82, 83], 27 yielded some concerns [32, 33, 37–39, 41, 43, 44, 47–49, 51, 53–55, 57–60, 64, 66–72] and four had a high risk of bias [31, 56, 61, 71]. In particular, one study [71] had a high risk of bias due to deviations from intended interventions, two studies [56, 61] had a high risk of bias due to bias in measurement of the outcome, and one study [31] had a high risk of bias due to both deviations from intended interventions and bias in measurement of the outcome.

**FIGURE 3:**
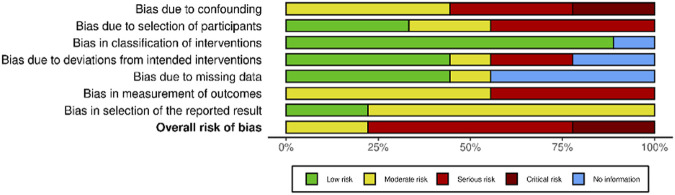
Risk of bias in non-randomized studies of interventions (ROBINS-I). Advancing Mammographic Screening Among Underserved Groups: A Systematic Review and Meta-Analysis of Intervention Strategies to Increase Breast Cancer Screening Uptake, Belgium, 2025.

Among non-randomized studies appraised with the ROBINS-I tool (Figure 3), no studies had a low risk of bias, two yielded some concerns [74, 80], five had a serious risk of bias [73, 75, 76, 78, 79] and two had a critical risk of bias [77, 81]. Because of the frequent serious and critical risk of bias detected among non-randomized studies, as well as the high variability in terms of study design, settings and populations, a meta-analysis of non-randomized studies was not performed.

The full risk of bias assessment is available at Supplementary Figures S14, S15.

### Meta-Analyses

**FIGURE 4:**
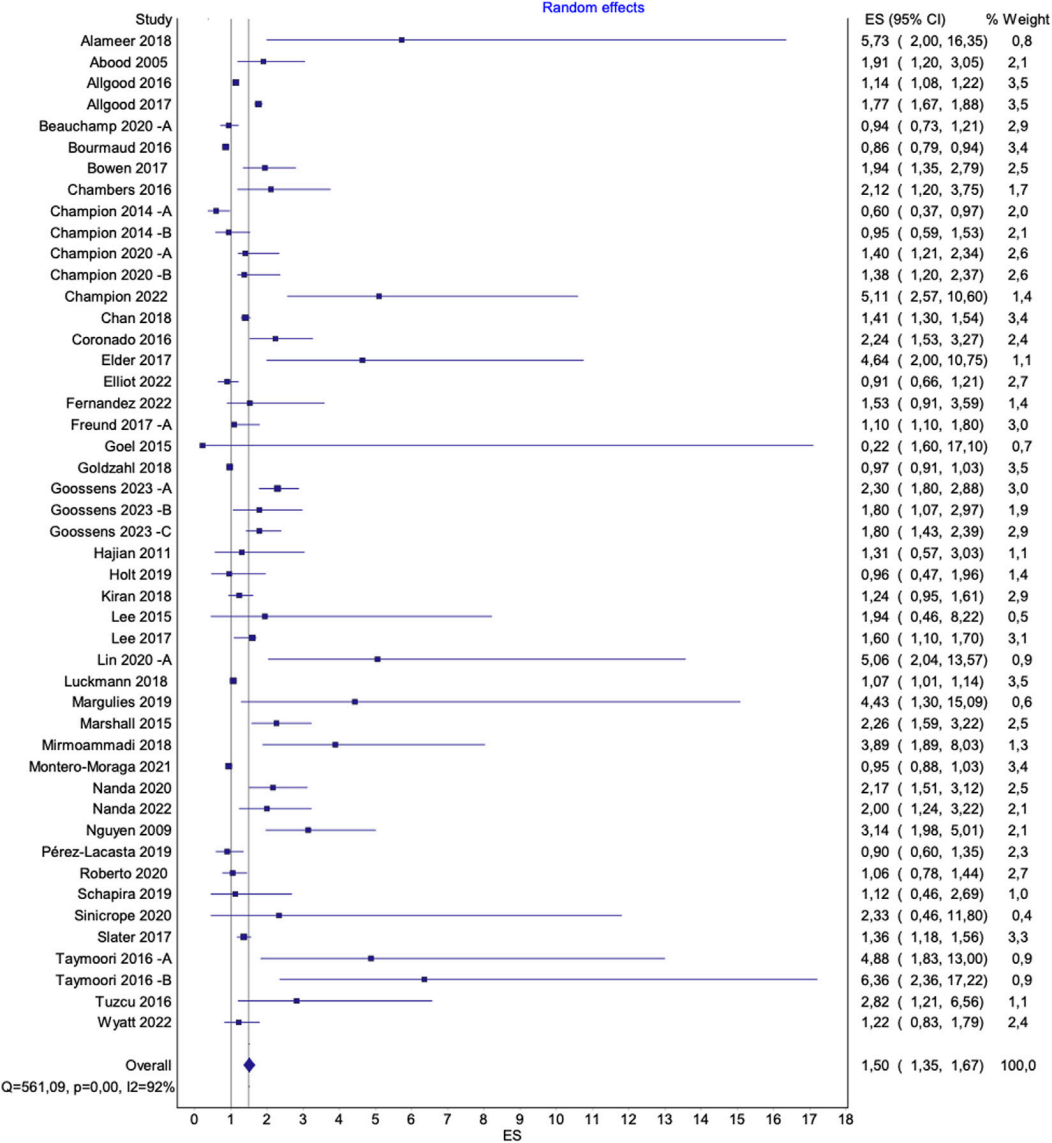
Overall pooled effect of interventions to increase mammography uptake. Advancing Mammographic Screening Among Underserved Groups: A Systematic Review and Meta-Analysis of Intervention Strategies to Increase Breast Cancer Screening Uptake, Belgium, 2025.

**FIGURE 5:**
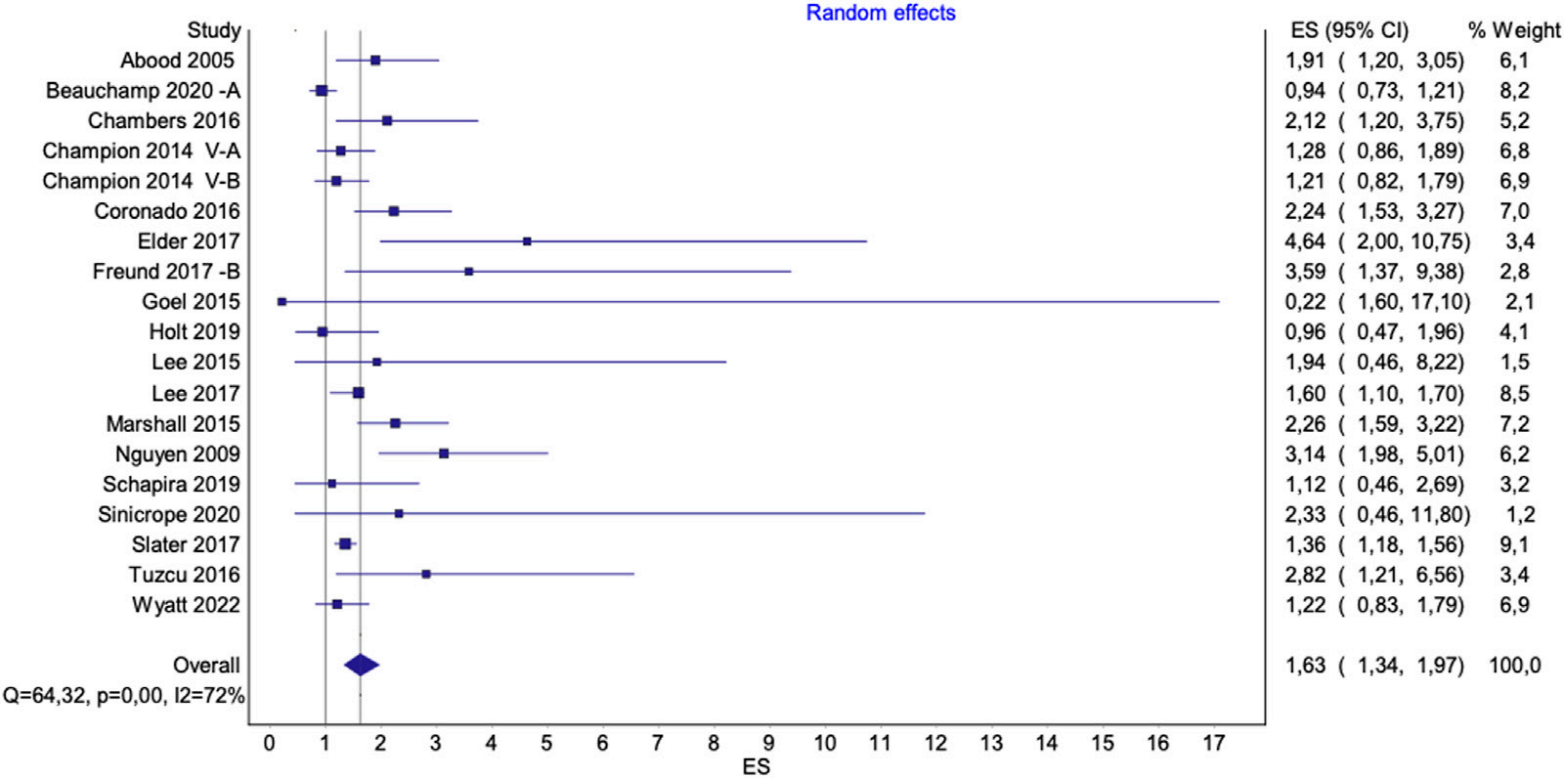
Pooled effect of interventions to increase mammography uptake among underserved groups. Advancing Mammographic Screening Among Underserved Groups: A Systematic Review and Meta-Analysis of Intervention Strategies to Increase Breast Cancer Screening Uptake, Belgium, 2025.

The pooled effects of interventions to increase the uptake of BC screening overall, and among underserved groups (as a subgroup), are presented in Figures 4, 5, respectively. Randomized studies investigating the effect of interventions to increase mammography uptake [31–35, 37, 39, 40, 42–44, 46, 48–54, 56–62, 65–67, 71–79, 81–83], repeated mammography [70], and combined indicators for either scheduled or obtained mammography [36, 47], were included in the meta-analysis.

#### Impact of Outliers and Risk of Bias on Result Interpretation

Two studies (Alizadeh-Sabeg et al. [33] and Kim et al. [53]) were identified as outliers due to extreme ORs resulting from zero events in the control groups, leading to off-scale estimates. These studies also had a moderate risk of bias with concerns related to the selection of reported results and contributed minimally to the overall effect size (each with a relative weight of 0.1%). Given these factors, they were excluded from subgroup and sensitivity analyses, and the main results presented in the manuscript reflect the pooled effect size without them. Forest plots including these studies are provided in the Supplementary Materials.

Additionally, a leave-one-out analysis was conducted to assess the impact of a third outlier (Beauchamp et al. [36] B). This study had a low risk of bias and a higher weight of 1%, which justified its inclusion in all analyses. To assess its impact on the overall effect size, results are presented both with and without this study.

The figures presented in the main text (Figures 4, 5) exclude all three outliers for visualization purposes. However, full figures including Beauchamp et al. [36] B are available in the Supplementary Materials.

#### Effectiveness of interventions for Increasing BC Screening Uptake

##### Overall

The pooled effects of interventions to increase mammography uptake overall, compared to standard care, showed significant results [OR 1.57 (95% CI 1.41–1.75), p < 0.0001; I^2^ = 92%] (Supplementary Figure S1A). Excluding two outliers (Alizadeh-Sabeg et al. [33] and Kim et al. [53]) with a relative weight of 0.1% each, did not notably alter the overall pooled effect [OR 1.55 (95% CI 1.39–1.73), p < 0.0001; I^2^ = 92%] (Supplementary Figure S1B). However, a leave-on-out analysis excluding a third outlier (Beauchamp et al. [36] B) resulted in a slightly lower effect size without reducing heterogeneity [OR 1.50 (95% CI 1.35–1.67), p < 0.0001; I^2^ = 92%] (Figure 4). A sensitivity analysis, excluding studies with a high risk of bias, did not substantially alter the pooled results [OR 1.56 (95% CI 1.39–1.76), p < 0.0001; I^2^ = 92%] (Supplementary Figure S1D).

##### Underserved Groups

The pooled effects of interventions that increased mammography uptake, in comparison to standard care, were higher for studies including underserved groups [OR 1.85 (95%CI 1.46–2.35), p < 0.0001; I^2^ = 82%] (Supplementary Figure S2A). Excluding the outlier study by Kim et al. [53] did not notably alter the overall pooled effect [OR 1.81 (95%CI 1.43–2.28), p < 0.0001; I^2^ = 82%] (Supplementary Figure S2B). When the study of Beauchamp et al. [36] (B) was excluded from the analysis, it resulted in a lower effect size, yet slightly reducing heterogeneity [OR 1.63 (95%CI 1.34–1.97), p < 0,0001; I^2^ = 72%] (Figure 5). A sensitivity analysis, excluding studies with a high risk of bias, did not substantially alter the results [OR 1.80, 95% CI 1.36–2.39, p < 0.0001; I^2^ = 84%] (Supplementary Figure S2D).

#### BC Screening Uptake by Type of Intervention

##### Educational Interventions

Educational interventions were among the most effective for increasing mammography uptake, with an OR of 2.61 (95% CI 1.78–3.82), p < 0.0001; I^2^ = 70% (Supplementary Figure S3A). In underserved groups, educational interventions also showed significant effects [OR 2.04 (95% CI 1.29–3.21), p < 0.0001; I^2^ = 73%] (Supplementary Figure S3B).

In this group, a sensitivity analysis was not performed as no studies were identified as having a high risk of bias.

##### Navigation Services

Navigation services significantly increased uptake as well [OR 2.10 (95% CI 1.61–2.73), p < 0.0001; I^2^ = 91%] (Supplementary Figure S4A), with similar results for underserved groups [OR 2.00 (95% CI 1.35–2.96), p < 0.0001; I^2^ = 90%] (Supplementary Figure S4D).

Excluding Beauchamp et al. [36] (B) reduced the effect size both overall [OR 1.84 (95% CI 1.45–2.33), p < 0.0001; I^2^ = 88%] (Supplementary Figure S4B) and for underserved groups [OR 1.62 (95%CI 1.22–2.16), p < 0.0001] (Supplementary Figure S4E). Instead, the sensitivity analysis excluding studies with a high risk of bias yielded results with higher effect size [OR 2.17, 95% CI 1.62–2.91, p < 0.0001; I^2^ = 91% (Supplementary Figure S4C); in underserved groups OR 2.10 (95%CI 1.24–3.54), p < 0.0001; I^2^ = 91% (Supplementary Figure S4F)].

##### Telephonic Interventions

Telephonic interventions were effective in increasing mammography uptake [OR 1.76 (95% CI 1.33–2.34), p < 0.0001; I^2^ = 88%] (Supplementary Figure S5A), especially in underserved groups [OR 2.66 (95% CI 1.42–4.98), p < 0.0001; I^2^ = 69%] (Supplementary Figure S5D).

Excluding Beauchamp et al. [36] (B) resulted in a lower effect size [OR 1.40 (95%CI 1.13–1.72), p < 0.0001; I^2^ = 88% (Supplementary Figure S5B); for underserved groups OR 1.65 (95%CI 1.24–2.19), p > 0.05; I^2^ = 42%] (Supplementary Figure S5E). A sensitivity analysis excluding studies with a high risk of bias yielded results with higher effect size [OR 1.89 (95% CI 1.33–2.70), p < 0.0001; I^2^ = 90% (Supplementary Figure S5C); for underserved groups OR 2.94 (95%CI 1.33–6.51), p < 0.0001; I^2^ = 91% (Supplementary Figure S5F)].

##### Reminders

Reminders also showed increased odds of mammography uptake [OR 1.64 (95% CI 1.34–1.99), p < 0.0001; I^2^ = 96%] (Supplementary Figure S6A), especially for underserved groups [OR 3.39 (95% CI 1.13–10.21), p < 0.0001; I^2^ = 95%] (Supplementary Figure S6C).

Excluding Beauchamp et al. [36] (B) resulted in a lower effect size [OR 1.47 (95% CI 1.22–1.76), p < 0.0001; I^2^ = 94%] (Supplementary Figure S6B). Notably, results for underserved groups were no longer statistically significant when Beauchamp et al. [36] (B) was excluded [OR 1.81 (95% CI 0.80–4.09), p > 0.05; I^2^ = 91%] (Supplementary Figure S6D).

A sensitivity analysis was not performed as no studies had a high risk of bias.

##### Invitation Letters

Invitation letters were generally effective [OR 1.44 (95% CI 1.20–1.72), p < 0.0001; I^2^ = 97%] (Supplementary Figure S7A).

A sensitivity analysis excluding high-risk bias studies did not substantially alter the results [OR 1.40 (95% CI 1.18–1.67), p < 0.0001; I^2^ = 96%] (Supplementary Figure S7B). The pooled estimates for underserved groups were not statistically significant.

##### Culturally Sensitive Interventions

For studies involving underserved groups, culturally sensitive interventions were highly effective [OR 1.98 (95% CI 1.19–3.29), p < 0.0001; I^2^ = 73%] (Supplementary Figure S8A). All studies retrieved in this category involved underserved groups.

As no studies had a high risk of bias, a sensitivity analysis was not performed.

The pooled estimates for studies investigating linguistically adapted interventions, decision aids, smartphone-based interventions, digital-based educational interventions and printed materials were not statistically significant. Due to the paucity of results, it was not possible to perform meta-analyses of studies investigating health consultations with a physician, the mediation of a family member, and interventions involving economic support. Complete forest and funnel plots are available in the Supplementary Materials.

#### Secondary Outcomes

Among 53 studies investigating the effect of interventions to increase the uptake of BC screening, 18 reported the effect of such intervention on secondary outcomes such as breast self-examination (BSE) and breast-awareness practices (BAP) [32, 38, 47, 51, 64, 74] and clinical breast examination (CBE) with a physician [44, 47, 51, 60, 71, 74].

For these outcomes, interventions also had a positive effect with ORs ranging between 1.33 and 6.36, and between 2.25 and 9.63, for BSE/BAP and for CEB, respectively. However, because of the limited number of studies available as well as the heterogeneity among them, a meta-analysis was not conducted. Study characteristics are reported in Supplementary Tables S3, S4.

### Certainty of Study Findings

The GRADE assessment revealed moderate certainty in the effectiveness of interventions for increasing BC screening uptake. The certainty was downgraded due to unexplained heterogeneity. The summary table is available at Supplementary Table S2.

## Discussion

### Main Findings

This review highlights the significant impact of interventions for increasing mammography uptake. Overall, these could increase uptake by 55%, with an even greater effect of 80% among underserved groups. This indicates that while underserved groups participate less in cancer screening, these groups have great potentials for improved participation.

Educational interventions emerged as the most effective strategy for increasing BC screening uptake, nearly tripling the odds of participation overall. These interventions were primarily delivered in person through lectures, workshops, and events. For example, a study featured a “Pink Party” (a community event in a festive setting) where participants learned about the importance of BC screening from BC survivors and medical professionals. Encounters with trained community members, such as *promotoras*, also offer personalized education and support, addressing individual concerns and encouraging health behaviors based on social norms. Additionally, digital approaches, such as videos shown in hospital waiting areas, help explain the BC screening process and improve comfort levels in clinical settings.

Navigation services have also proven highly effective, doubling the odds of BC screening uptake both overall and among underserved groups. These services offer comprehensive support to guide individuals through the healthcare system. For instance, navigators build collaborative relationships with participants, identify their needs, and work to overcome barriers. If transportation is an issue, the navigator can arrange for transportation support. Navigators might also accompany participants to their screenings when necessary. In another example, navigators provided participants with educational materials and coached them on questions to ask their healthcare providers, enhancing their confidence and engagement in the screening process. Indeed, other types of communication strategies about the BC screening program directed towards the target group proved highly effective.

Especially among underserved groups, reminders and telephonic interventions were highly effective, increasing the odds of participation by up to three times. For example, a follow-up reminder letter with a set date can be sent a few weeks after the initial invitation if no appointment has been scheduled. Reminders can be personalized by being sent in the participant’s preferred language, including a quote or signature from their own GP or other referral healthcare providers, or by providing simplified information about the BC screening process. Telephonic interventions also offer a personalized approach to encourage mammography uptake. In one study, participants received calls where trained healthcare workers employed a loss-framed strategy, highlighting the risks of undetected malignancies and emphasizing the high efficacy of mammograms in early detection. For instance, a caller might say, “By getting a mammogram now, you can catch any potential issues early, significantly increasing the chances of successful treatment.” Telephonic interventions can also lead to more comprehensive navigation services, where callers assist with appointment scheduling, address concerns, and coordinate other necessary services.

Finally, community-sensitive approaches tailored to the values, religious beliefs, and practices of specific groups have also proven highly effective. For example, religious institutions can play a role through community health advisors who provide culturally sensitive educational materials on BC screening. In a study involving Arab women, phone calls were used to address cultural barriers. To counter the belief that cancer is an immutable fate, callers referenced religious teachings that emphasize personal health responsibility, stating, “Both Muslim and Christian teachings highlight the importance of taking care of your health. Early detection through mammography can save lives.” These culturally sensitive conversations also tackled practical concerns, such as fear of pain, by explaining that discomfort lasts only a few seconds and suggesting scheduling the mammogram a week after menstruation to reduce sensitivity. Additionally, callers addressed concerns about the gender of the healthcare provider, reassuring participants that many facilities offer the option to choose a female doctor if preferred, thus respecting personal comfort and cultural preferences.

Evidence suggests that interventions are often more effective when implemented together [85]. By integrating the strategies highlighted in this article, multiple barriers could be addressed simultaneously, ensuring participants receive comprehensive support throughout the breast cancer screening process. For example, a program might begin with an invitation letter, followed by a reminder letter in the participant’s preferred language. If no response is received, a navigator could then follow up by phone, offering to schedule the appointment and providing logistical support as needed. However, further research is warranted to assess the combined effectiveness of these strategies and to determine their impact on BC screening rates.

While the majority of studies included in this meta-analysis did not explicitly assess the cost-effectiveness of the interventions, this aspect was occasionally discussed in a speculative or theoretical manner. For example, interventions requiring minimal personnel involvement, such as paper-based or digital letters and reminders, were often considered more likely to be cost-effective due to their low operational costs. Future research should explicitly evaluate the cost-effectiveness of these strategies to better inform their practical application and potential for widespread adoption.

### Strengths and Limitations

This study comprehensively reviews a broad range of interventions, providing a holistic view of strategies to increase BC screening uptake. By specifically examining the impact of interventions on underserved groups, the study addresses a critical gap in the literature, providing actionable insights for healthcare providers and policymakers.

The substantial sample size across the studies included in this review (161,141 individuals, with 14,720 from underserved groups) adds robustness to the findings and enhances the generalizability of the results.

According to the GRADE assessment, the findings are attributed with moderate certainty, indicating that the results are generally reliable. However, the study findings also exhibit unexplained heterogeneity, which is defined as the presence of variation in true effect sizes across different studies. The inclusion of studies conducted in diverse populations and settings, along with the use of comparators other than usual care, might contribute to this heterogeneity, necessitating cautious interpretation of the results.

To investigate the impact of specific studies, leave-one-out analyses were conducted. Three main outliers were identified. The studies by Alizadeh-Sabeg et al. [33] and Kim et al. [53] had minimal impact due to their low relative weight. In contrast, excluding the third outlier, Beauchamp et al. [36] (B), slightly lowered the overall effect size, suggesting some sensitivity to individual studies, without reducing heterogeneity. Sensitivity analyses excluding studies with a high risk of bias largely confirmed the reliability of the study results. A random-effects model was applied to account for unexplained heterogeneity.

### Comparison With Prior Work

Our review aimed to fill the temporal gap left after Agide et al.’s groundwork that included 22 studies published between 2004 and 2016 [21]. These reports were assessed for eligibility according to our selected inclusion criteria. Ultimately, 20 of these studies were included in our analysis, along with an additional 34 studies published between 2017 and 2023. While this study focused on the type of study by setting (individual-based interventions, community-based interventions, and interventions via religious, cultural promoters and lay workers), our study investigated the effectiveness of interventions by format and content (educational interventions, telephonic interventions, navigation services, linguistically adapted interventions, culturally sensitive interventions, digital-based educational intervention, smartphone-based interventions, decision aids, invitation letters, reminders, health consultations with physicians, printed materials, economic support, family member mediation), providing a more granular understanding of which specific strategies and formats are most effective in increasing participation, especially among underserved communities.

To our knowledge, this is the first recent meta-analysis on interventions to improve mammographic screening with emphasis on a diverse spectrum of underserved groups (eg. individuals with low-SES, underinsured or uninsured, with an immigrant background, belonging to an ethnic minority, or facing possible cultural or religious barriers). In fact, while other reviews on the topic have been published, these would focus exclusively on specific subgroups such as ethnic minority women [86], or women living in low-income and middle-income countries [87]. Our findings, in conjunction with insights from these existing resources, can guide the development of comprehensive strategies to enhance mammographic screening participation among underserved groups.

### Real-World Application

This review is part of the project “ENTER: Equity in breast cancer screening in Flanders” [88]. In line with its overarching goal of addressing disparities in BC screening participation among women with low socioeconomic status in Flanders (Belgium), a practical objective of this review was to pinpoint interventions - and their features - that could be seamlessly integrated into the existing Flemish BC screening program, considering its logistical, contextual and social context.

Considering costs, practicality and impact, a reminder letter with culturally and linguistically adapted content emerged as the most promising option for tryout in the ENTER project and is in the meantime being implemented in a pilot RCT.

## Conclusion

This review underscores that targeted interventions can significantly reduce disparities in healthcare access, particularly for underserved populations. Implementing educational initiatives, navigation services, personalized reminders, and culturally sensitive approaches has the potential to substantially increase participation in BC screening programs.

Underserved groups often show lower BC screening rates, which can result in delayed diagnoses and more advanced, less treatable cancer stages. This not only imposes a considerable burden on individuals and their families but also necessitates more intensive and costly treatments associated with late-stage cancer. Increasing screening uptake in these populations can help address these challenges and improve the efficiency of healthcare resource use.

This review offers practical insights for policymakers and stakeholders responsible for implementing BC screening programs. By synthesizing evidence on strategies that reduce screening barriers and promote health equity, these findings can help guide improvements in program design and inform future healthcare and policy planning.
