# Supplementary material for: Advancing Mammographic Screening Among Underserved Groups: A Systematic Review and Meta-Analysis of Intervention Strategies to Increase Breast Cancer Screening Uptake
Source: Public Health Rev. 2025 Apr 4;46:1607873. doi: 10.3389/phrs.2025.1607873 (PMC12006734; doi:10.3389/phrs.2025.1607873)
Supplement: Supplementary file 1 [file DataSheet1.pdf]

Supplementary Materials Ferrari et al. 2024

<u>Methodological notes</u>

1) Impact of outliers and risk of bias on result interpetration

Two studies (Alizadeh-Sabeg et al. 2021 and Kim et al. 2022) were identified as outliers due to extreme ORs resulting from zero events in the control groups, leading to off-scale estimates. These studies also had a moderate risk of bias and contributed minimally to the overall effect size (each with a relative weight of 0.1%). Given these factors, they were excluded from subgroup and sensitivity analyses, and the main results presented in the manuscript reflect the pooled effect size without them.
Additionally, a leave-one-out analysis was conducted to assess the impact of a third outlier (Beauchamp et al. 2020 B). This study had a low risk of bias and a higher weight of 1%, which justified its inclusion in all analyses. To assess its impact on the overall effect size, results are presented both with and without this study.
The figures available in the main text (Figures 4 and 5) exclude all three outliers for visualization purposes. However, full figures are presented in the Supplementary Materials.

2) Sensitivity analysis

Sensitivity analyses were conducted where possible and for each subgroup to assess the robustness of the results. These analyses specifically accounted for the potential influence of studies with a high risk of bias, as assessed using the ROB-2 tool.

3) Subgroup Analyses

Subgroup analysis were performed for both population type (overall population, underserved) and intervention type (educational interventions, telephonic interventions, navigation services, invitation letters, reminders, linguistically adapted interventions, culturally sensitive interventions, digital-based educational interventions, smartphone-based interventions, decision aids, printed materials).
Where possible studies presenting results for multiple interventions or populations were included separately in the analysis (labelled in letters) and were therefore presented in distinct forest plots. If a study used more than one comparator, the comparator closest to standard care was chosen. For studies with different follow-up periods, we considered the longest follow-up. In particular:

Beauchamp 2020 A and B: This study had two separate interventions with distinct control groups. Group A received an invitation letter, while Group B underwent a telephonic intervention.

Champion 2014 A and B, V-A and V-B: This study included two interventions with a single control group, which was split into two for the analysis. Group A received media educational interventions and Group B received telephonic interventions. Results for high/average SES groups were pooled in the meta-analyses for the overall population (Champion 2014 A and B). Results for low SES groups were pooled in the meta-analyses for underserved populations (Champion 2014 V-A and V-B).

Champion 2020 A and B: This study included two interventions with a single control group, divided into two for the analysis. Group A received a decision aid, while Group B received a telephonic intervention.

Freund 2017 A and B: Conducted in Israel, this study investigated two subgroups: Ultra-Orthodox Jews and Palestinian Arabs. Results for the entire study population were pooled in the meta-analyses for the overall population, while results for the Palestinian Arabs group were pooled in the meta-analyses for underserved populations.

Goossens 2023 A, B, and C: This study had three clusters, each with different control groups (A Mobile unit among women with a history of nonattendance, B Hospital-based unit among women with a history of non-attendance, C Hospital-based unit among women with a history of irregular attendance).

Lin 2020 A and B: This study included two interventions with a unique control group. The control group was considered too small to divide, so results for the two interventions were pooled separately. Results for Group A (telephonic intervention, navigation) were pooled overall, and specifically in the telephonic intervention and navigation meta-analyses. Results for Group B (invitation letter, reminder) were pooled in the meta-analyses for invitation letters and reminders.

Taymoori 2016 A and B: This study included two interventions with a single control group, divided into two for the analysis. Group A received an educational intervention based on the Health Belief Model (HBM), while Group B received an educational intervention based on the Theory of Planned Behavior (TPB).

A full list of intervention and comparators is presented in Supplementary Materials Table 5.

4) Publication Bias

Funnel plots were generated when applicable (i.e., when there were ≥10 studies per outcome or subgroup) to visually assess the potential for publication bias.

All adjustments were made in accordance with the Cochrane Handbook for Systematic Reviews of Interventions guidelines:

Higgins JPT, Thomas J, Chandler J, Cumpston M, Li T, Page MJ, Welch VA. Cochrane Handbook for Systematic Reviews of Interventions - 9.3.4 Repeated observations on participants. https://handbook-5-1.cochrane.org/v5.0.2/chapter_9/9_3_4_repeated_observations_on_participants.htm; Higgins JPT, Thomas J, Chandler J, Cumpston M, Li T, Page MJ, Welch VA. Cochrane Handbook for Systematic Reviews of Interventions - 16.5.4 How to include multiple groups from one study. https://handbook-5-1.cochrane.org/chapter_16/16_5_4_how_to_include_multiple_groups_from_one_study.htm

<u>**Interventions to increase mammography uptake by population type**</u>

Figure 1A - Overall pooled effect of interventions to increase mammography uptake.

Forest plot 1A includes all identified studies investigating the effect of interventions to increase mammography uptake. Results from Alizadeh-Sabeg 2021 and Kim 2022, later identified as outliers (with a relative weight of 0,1% each) and excluded from further analyses, <u>are included in this plot</u>.

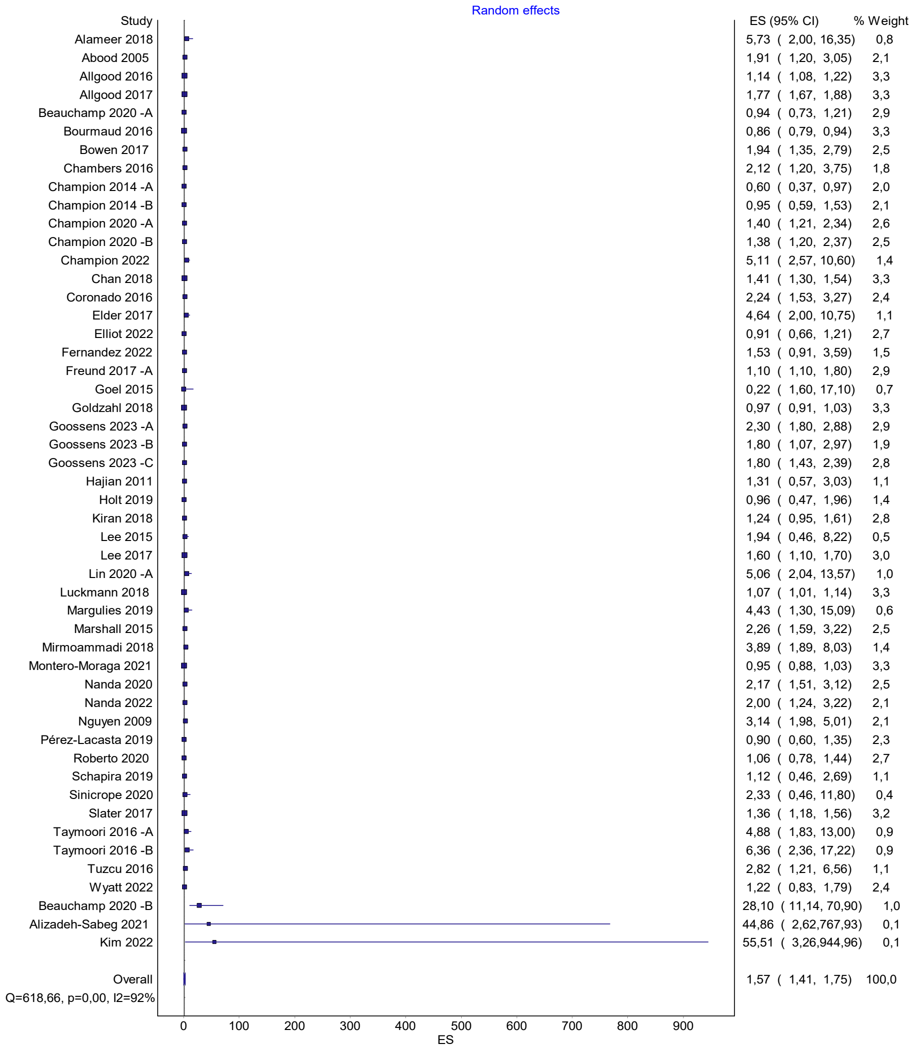

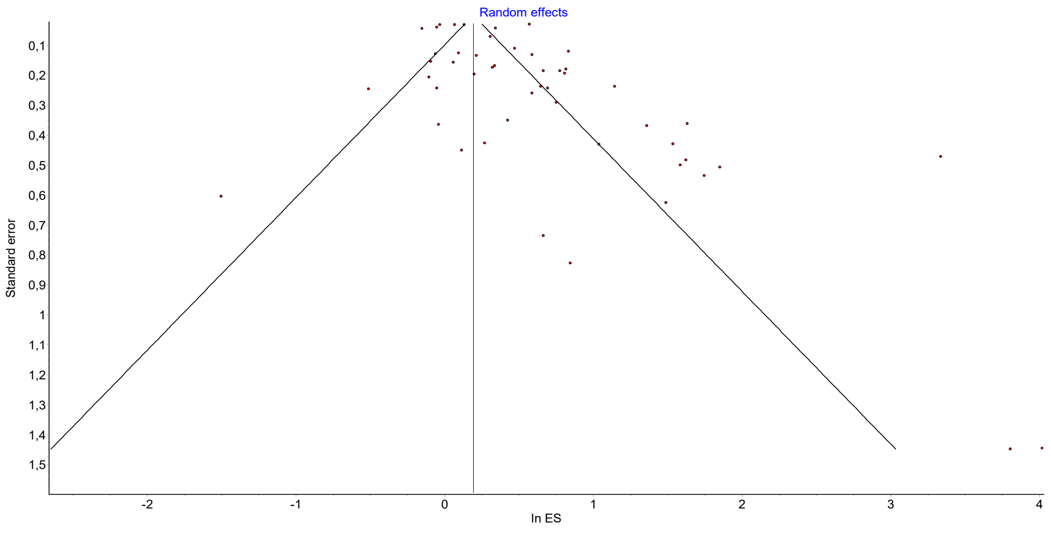

Figure 1B - Overall pooled effect of interventions to increase mammography uptake (excl. 2 outliers)

Forest plot 1B includes all identified studies investigating the effect of interventions to increase mammography uptake, except for results from Alizadeh-Sabeg 2021 and Kim 2022, identified as outliers (with a relative weight of 0,1% each) and excluded from further analyses.

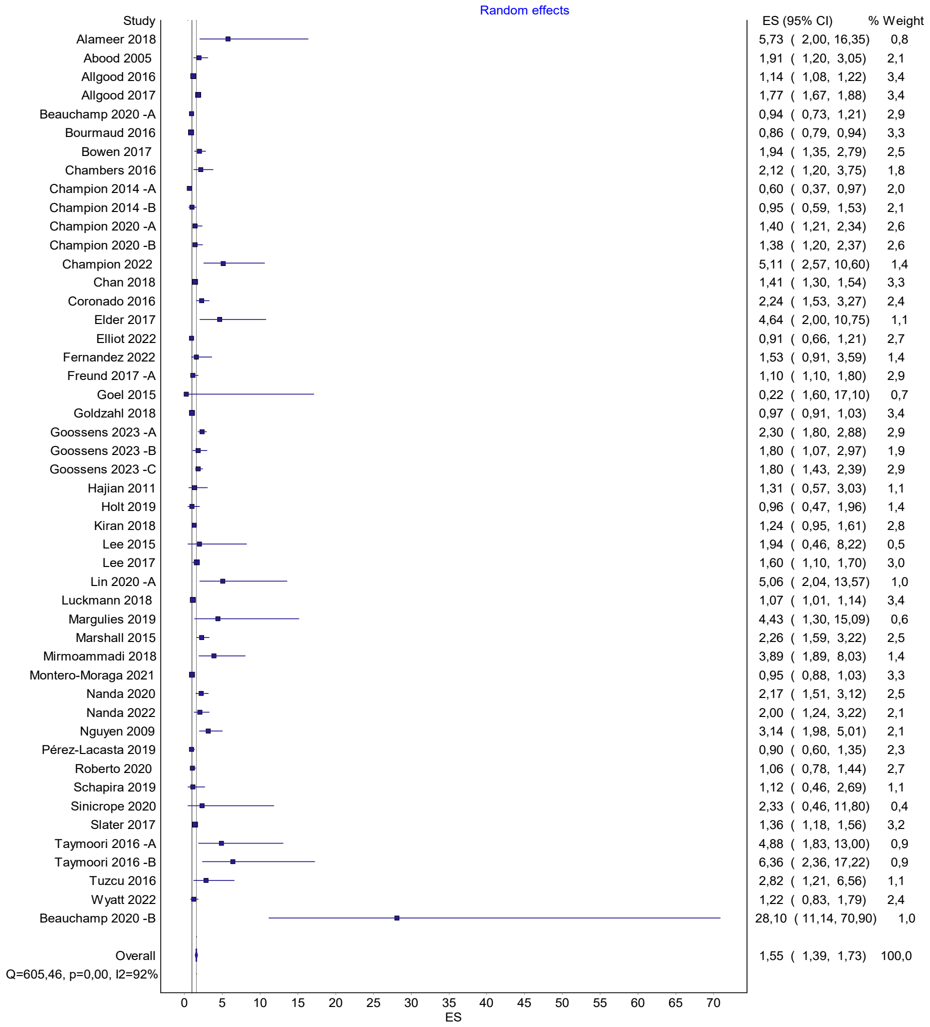

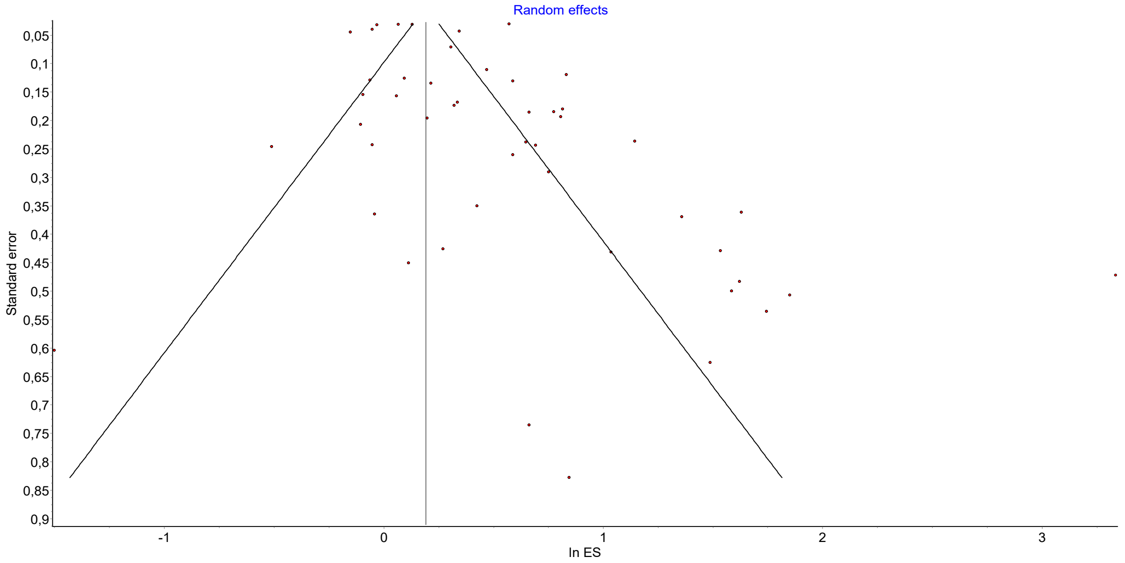

Figure 1C - Overall pooled effect of interventions to increase mammography uptake (excl. *Beauchamp 2020-B)*

Forest plot 1C includes identified studies investigating the effect of interventions to increase mammography uptake. In this case, results from Alizadeh-Sabeg 2021 and Kim 2022, identified as outliers (with a relative weight of 0,1% each) and Beauchamp 2020-B (with a relative weight of 1%) were excluded from the analysis.

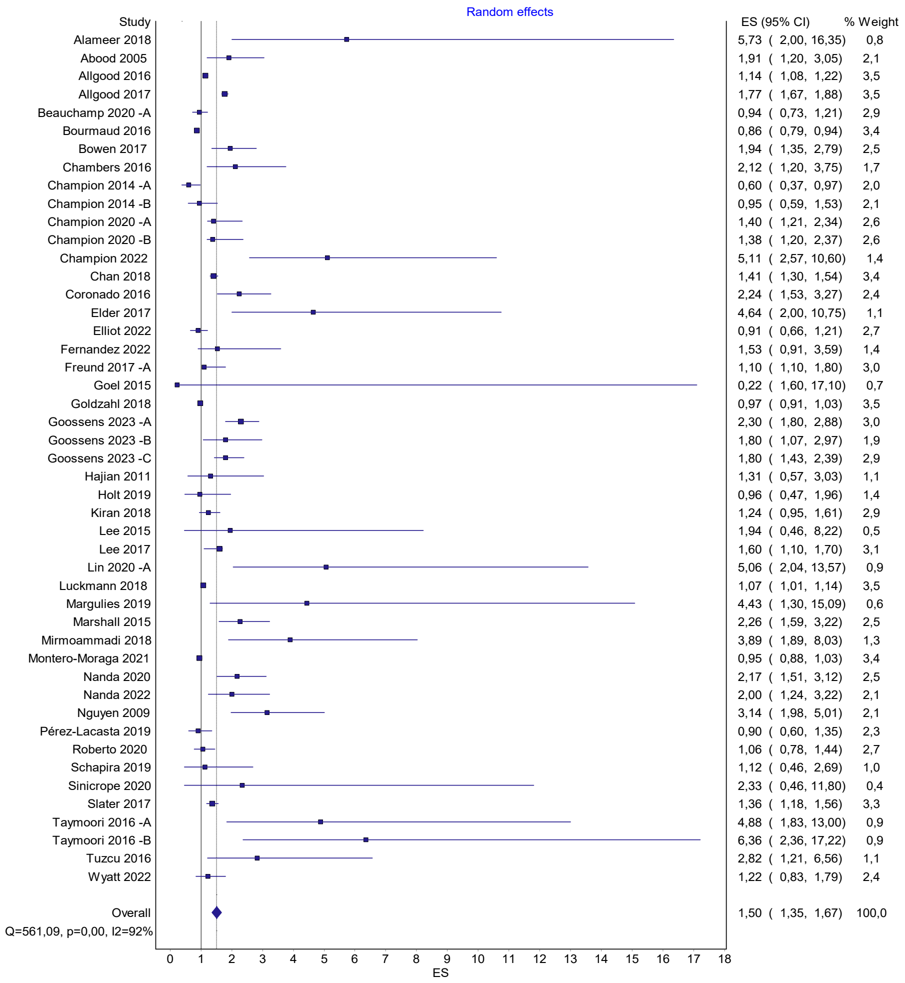

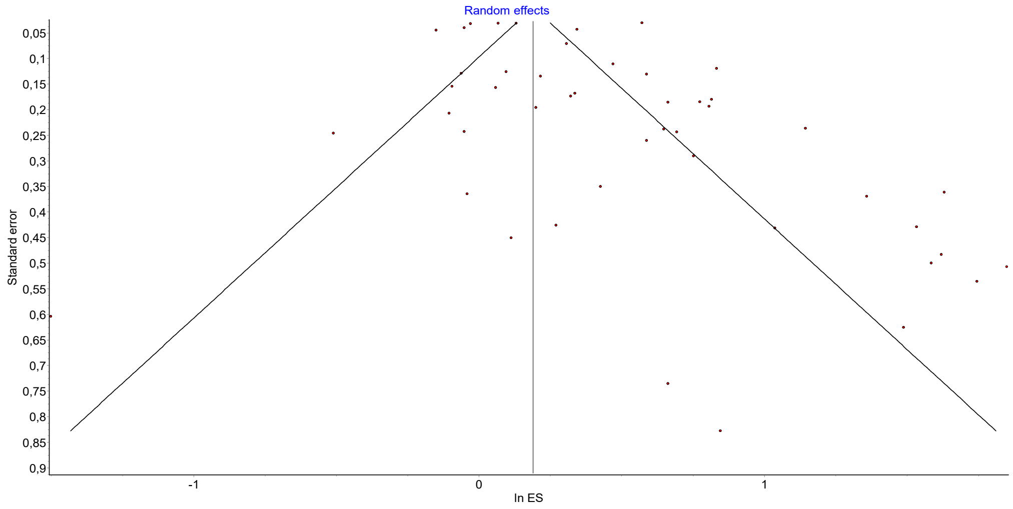

Figure 1D - Overall pooled effect of interventions to increase mammography uptake (sensitivity analysis)

Forest plot 1D includes identified studies investigating the effect of interventions to increase mammography uptake. In this case, results from Alizadeh-Sabeg 2021 and Kim 2022, identified as outliers (with a relative weight of 0,1% each) and studies judged to be at high risk of bias, are excluded.

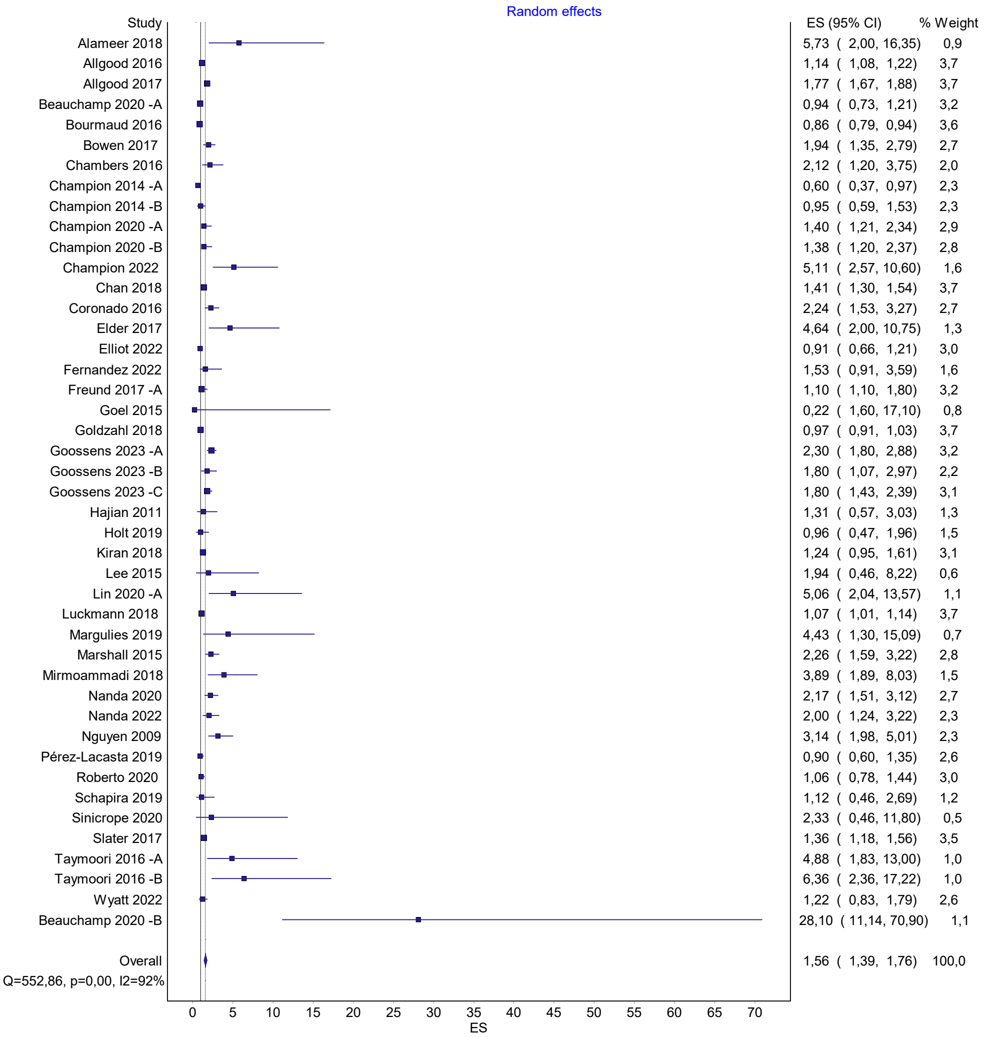

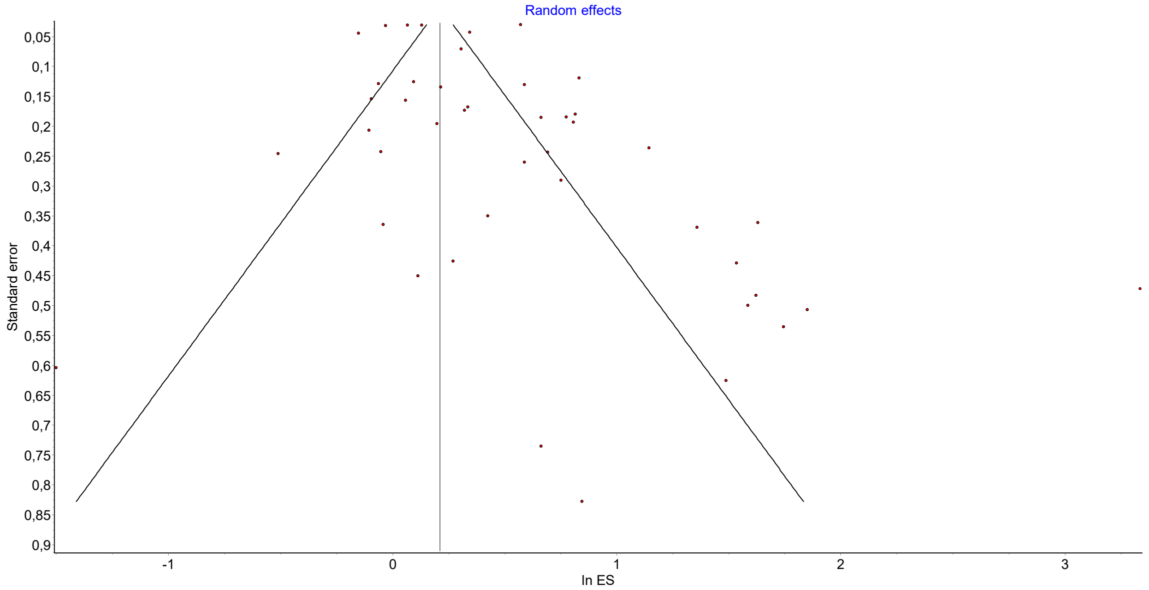

Figure 2A - Pooled effect of interventions to increase mammography uptake among underserved groups

Forest plot 2A includes all identified studies investigating the effect of interventions to increase mammography uptake among underserved groups. Results from Kim 2022, later identified as outlier (with a relative weight of 0,1%) and excluded from further analyses, ***are included in this plot.***

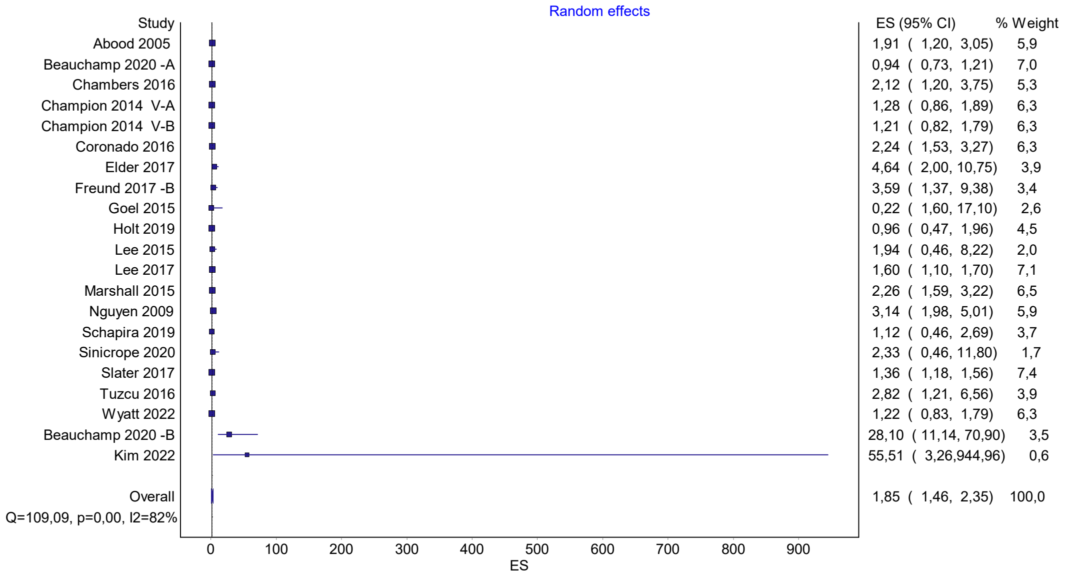

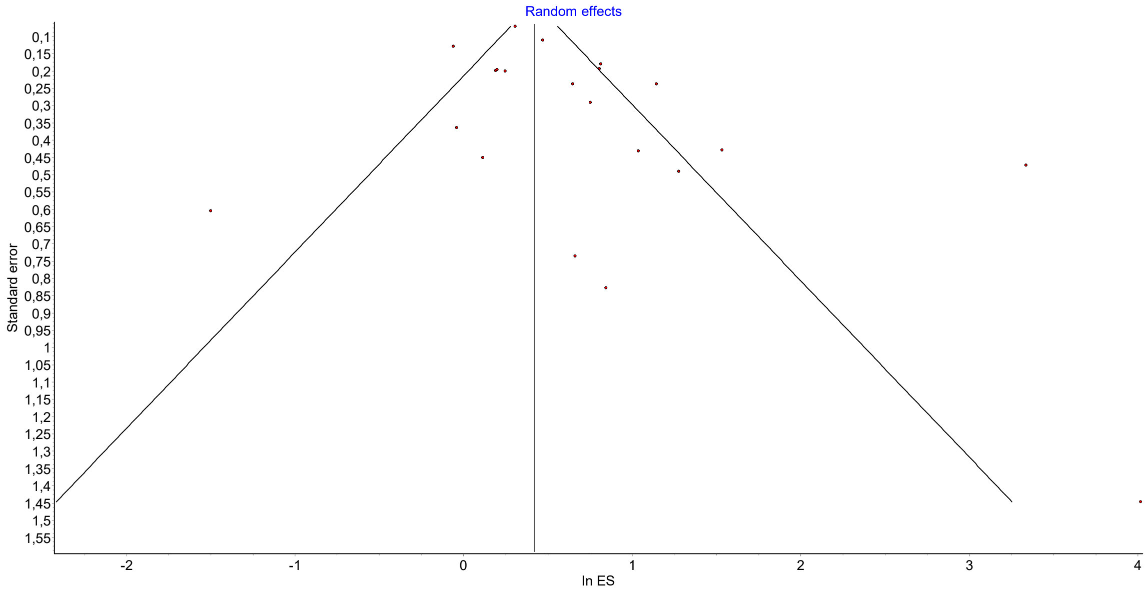

Figure 2B - Overall pooled effect of interventions to increase mammography uptake among underserved groups (excl. 1 outlier)

Forest plot 2B includes all identified studies investigating the effect of interventions to increase mammography uptake among underserved groups, except for results from Kim 2022, identified as outliers (with a relative weight of 0,1%) and excluded from further analyses.

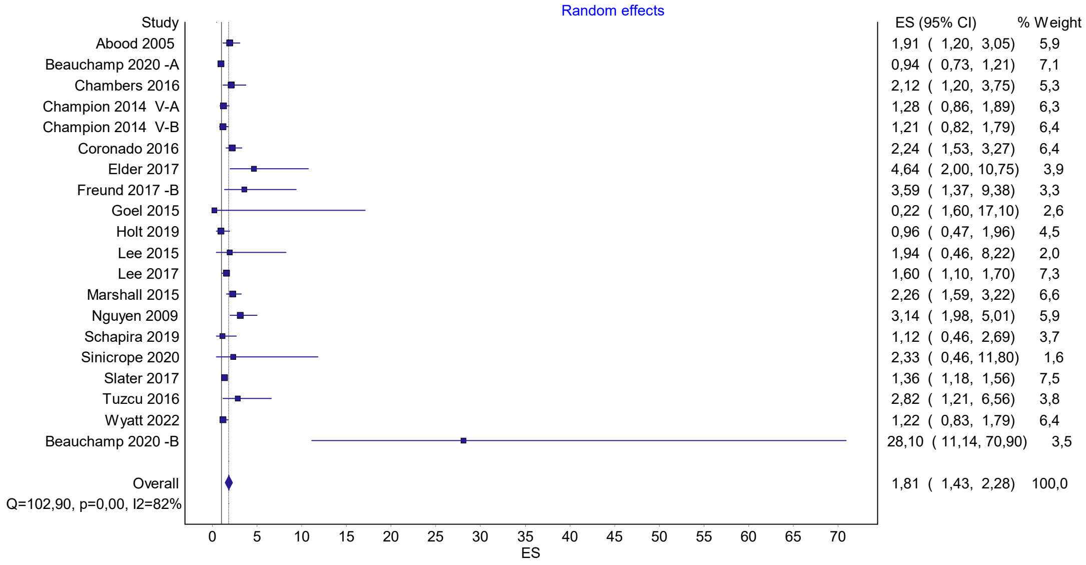

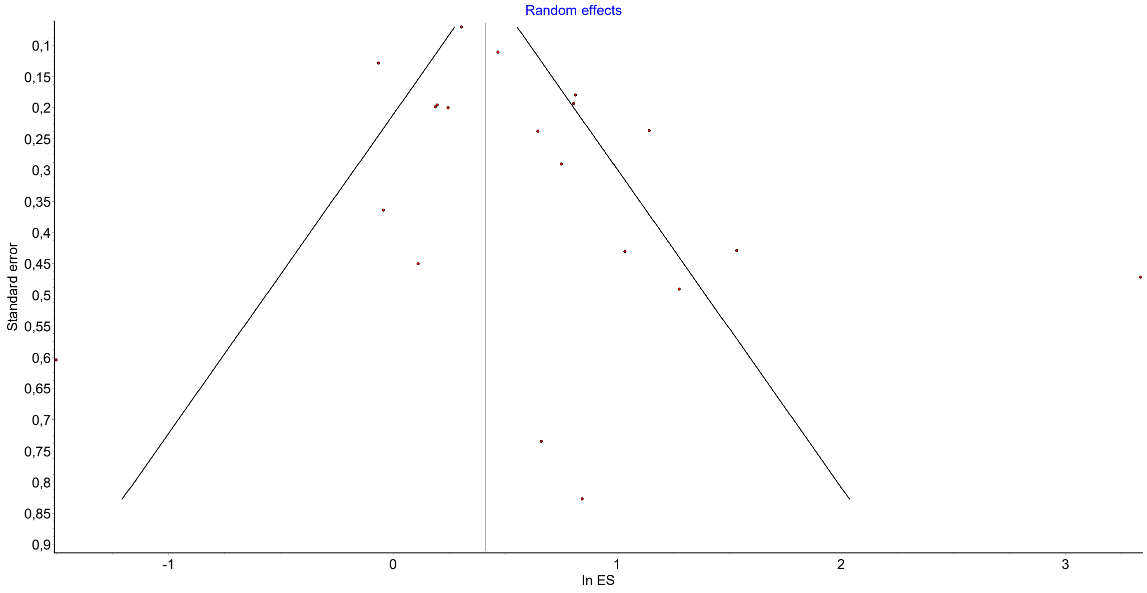

Figure 2C - Pooled effect of interventions to increase mammography uptake among underserved groups (excl. *Beauchamp 2020-B)*

Forest plot 2C includes identified studies investigating the effect of interventions to increase mammography uptake among underserved groups. In this case, results from Kim 2022 (with a relative weight of 0,1%) and Beauchamp 2020-B (with a relative weight of 1%) were excluded from the analysis.

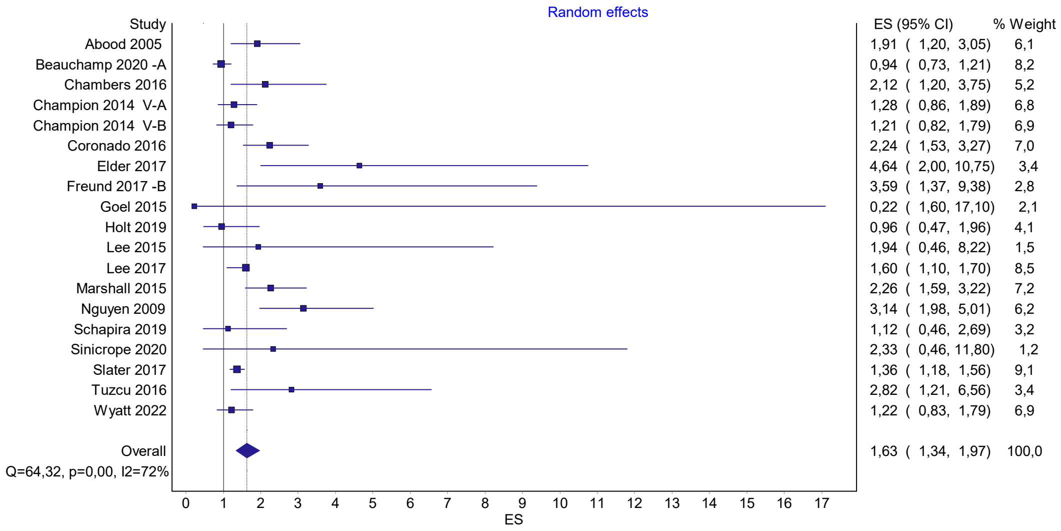

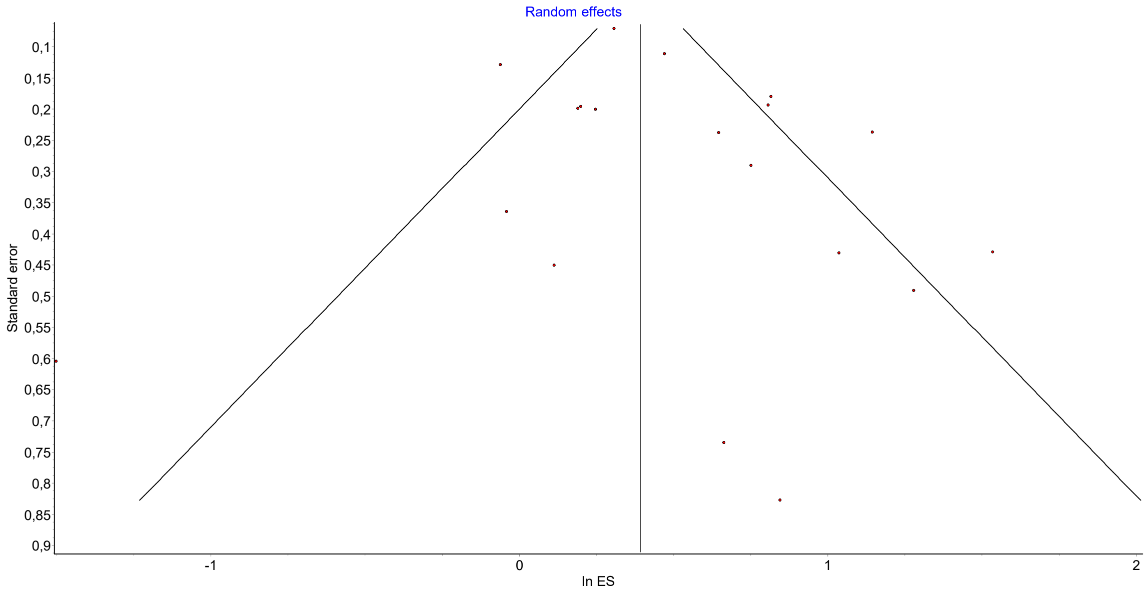

Figure 2D - Pooled effect of interventions to increase mammography uptake among underserved groups (sensitivity analysis)

Forest plot 2D includes identified studies investigating the effect of interventions to increase mammography uptake among underserved groups. In this case, results from Kim 2022, identified as outlier (with a relative weight of 0,1%) and studies judged to be at high risk of bias, are excluded.

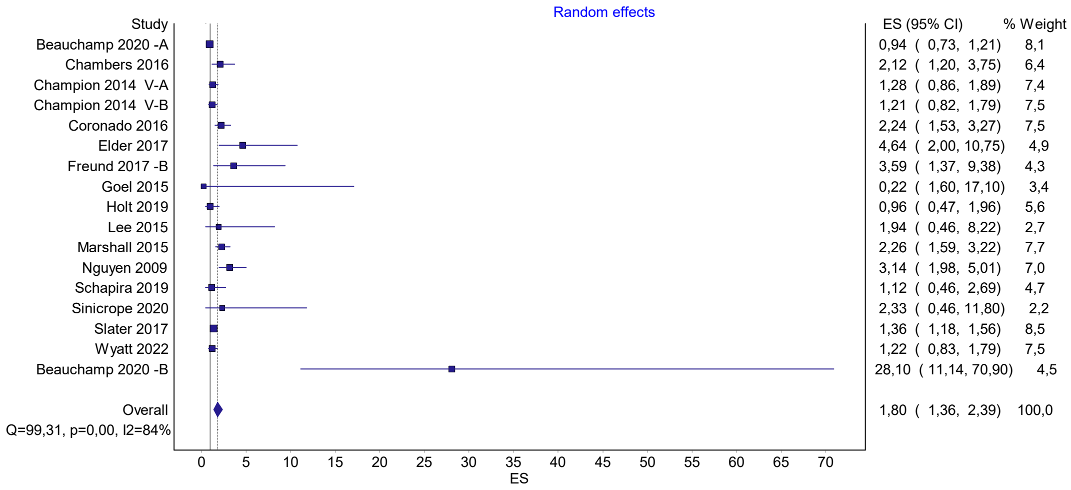

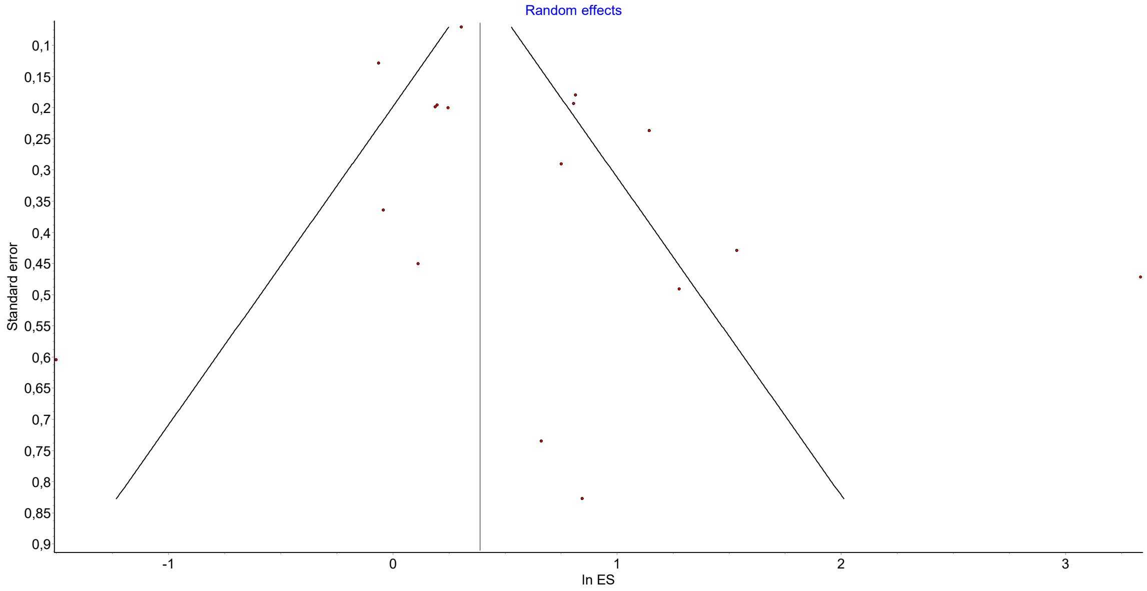

Interventions to increase mammography uptake by type of intervention

- **Educational interventions**

Figure 3A - Overall pooled effect of educational interventions to increase mammography uptake (excl. 2 outliers)

Forest plot 3A includes all identified studies investigating the effect of educational interventions to increase mammography uptake, except for results from Alizadeh-Sabeg 2021 and Kim 2022, identified as outliers (with a relative weight of 0,1% each) and excluded from further analyses.

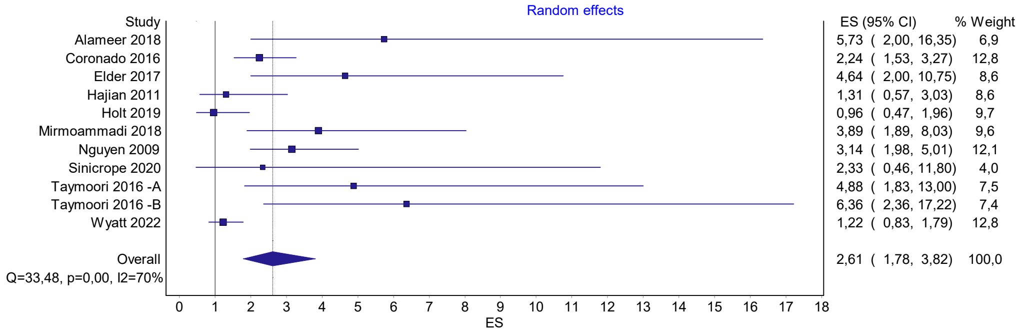

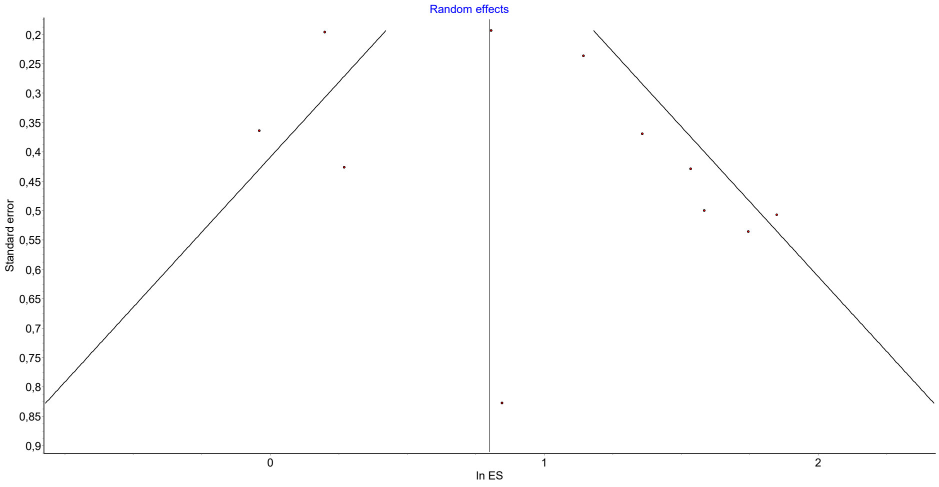

Figure 3B - Pooled effect of educational interventions to increase mammography uptake among underserved groups (excl. 1 outlier)

Forest plot 3D includes all identified studies investigating the effect of educational interventions to increase mammography uptake among underserved groups, except for results from Kim 2022, identified as outlier (with a relative weight of 0,1%), and excluded from further analyses.

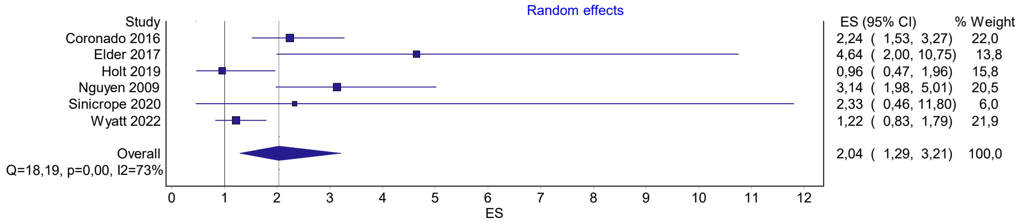

- **Navigation services**

Figure 4A - Overall pooled effect of navigation services to increase mammography uptake (excl. 1 outlier)

Forest plot 4A includes all identified studies investigating the effect of navigation services to increase mammography uptake, except for results from Kim 2022, identified as outliers (with a relative weight of 0,1%) and excluded from further analyses.

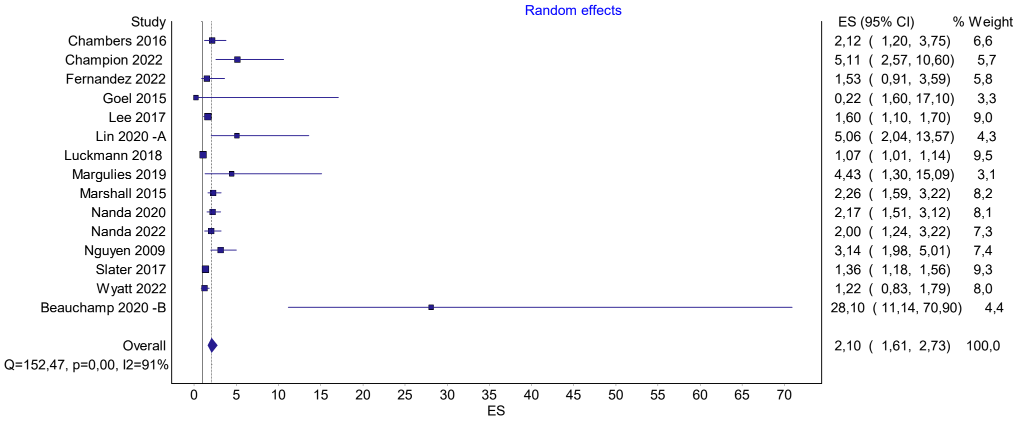

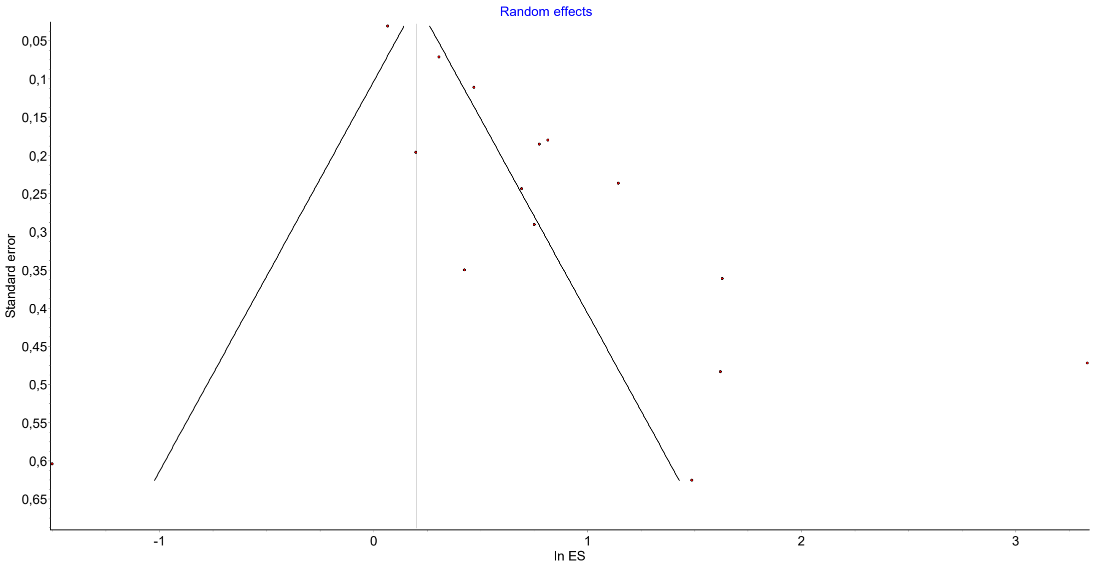

Figure 4B - Overall pooled effect of interventions to increase mammography uptake (excl. *Beauchamp 2020-B)*

Forest plot 4B includes identified studies investigating the effect of navigation services to increase mammography uptake.
In this case, results from Kim 2022, identified as outlier (with a relative weight of 0,1%), and Beauchamp 2020-B (with a relative weight of 1%) were excluded from the analysis.

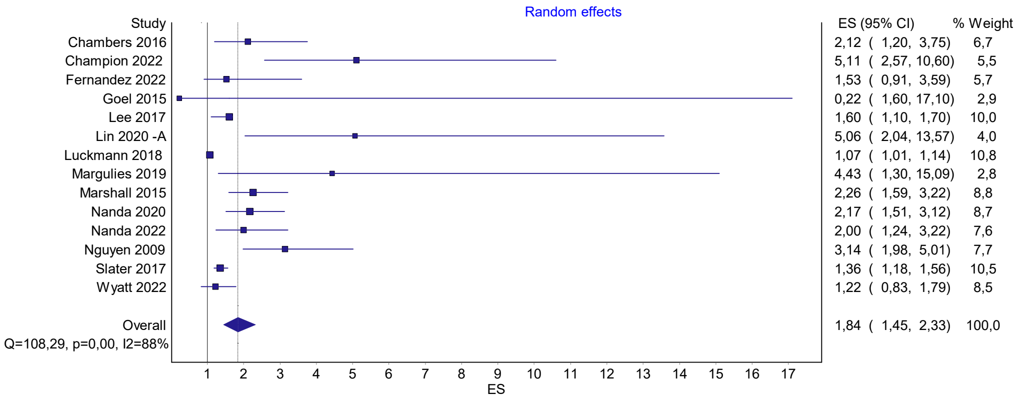

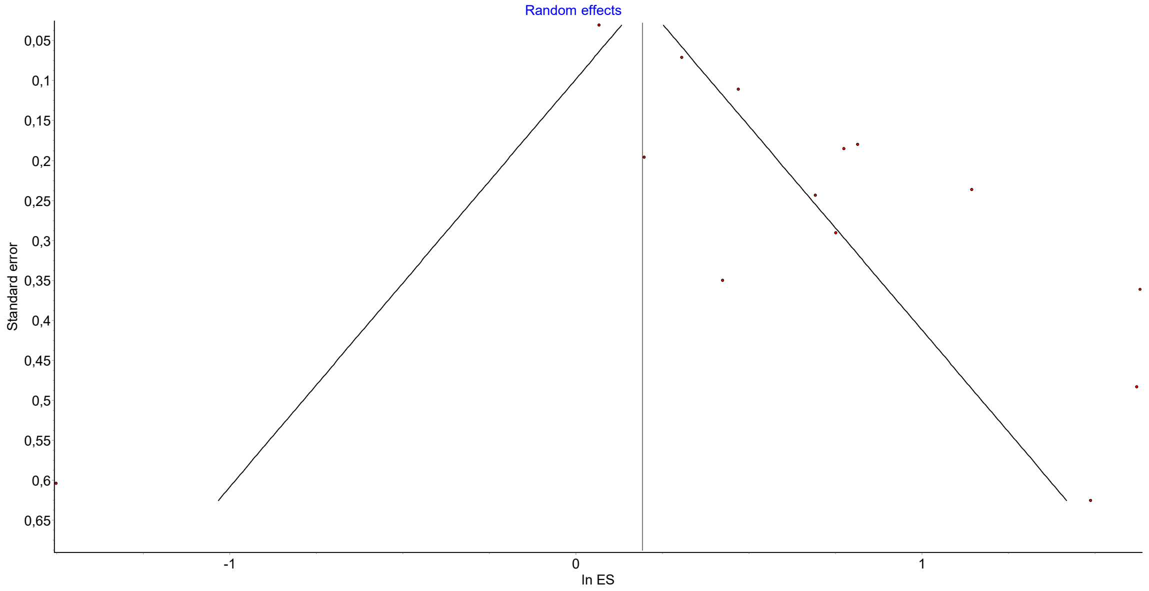

Figure 4C - Overall pooled effect of navigation services to increase mammography uptake (sensitivity analysis)

Forest plot 4C includes identified studies investigating the effect of navigation services to increase mammography uptake.
In this case, results from Kim 2022, identified as outlier (with a relative weight of 0,1%), and studies judged to be at high risk of bias, are excluded.

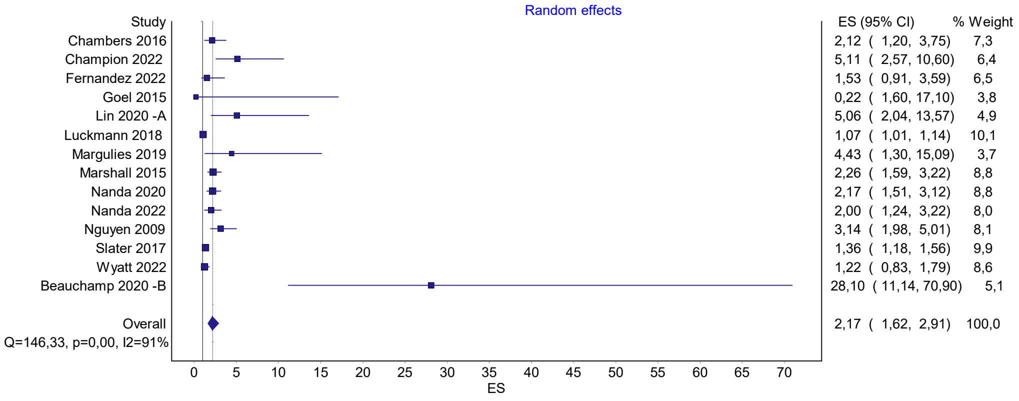

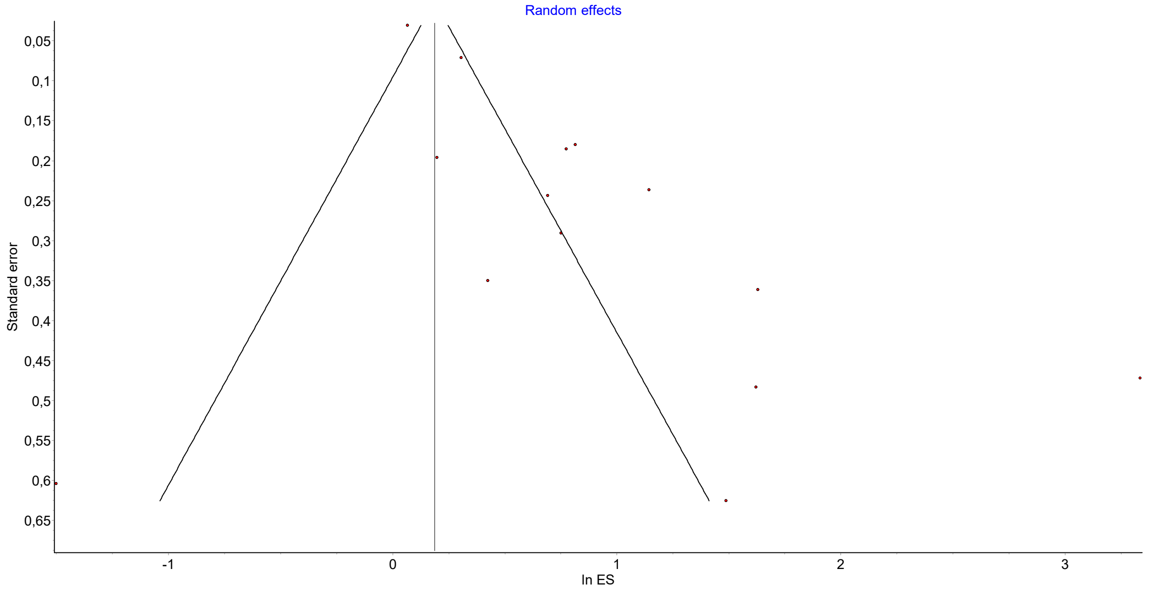

Figure 4D - Pooled effect of navigation services to increase mammography uptake among underserved groups (excl. 1 outlier)

Forest plot 4D includes all identified studies investigating the effect of navigation services to increase mammography uptake among underserved groups, except for results from Kim 2022, identified as outlier (with a relative weight of 0,1%) and excluded from further analyses.

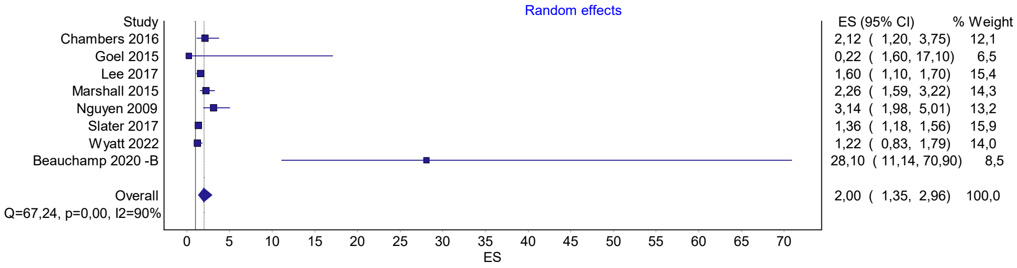

Figure 4E - Pooled effect of navigation services to increase mammography uptake among underserved groups (excl. *Beauchamp 2020-B)*

Forest plot 4E includes identified studies investigating the effect of navigation services to increase mammography uptake among underserved groups.
In this case, results from Kim 2022, identified as outlier (with a relative weight of 0,1%), and Beauchamp 2020-B (with a relative weight of 1%) were excluded from the analysis.

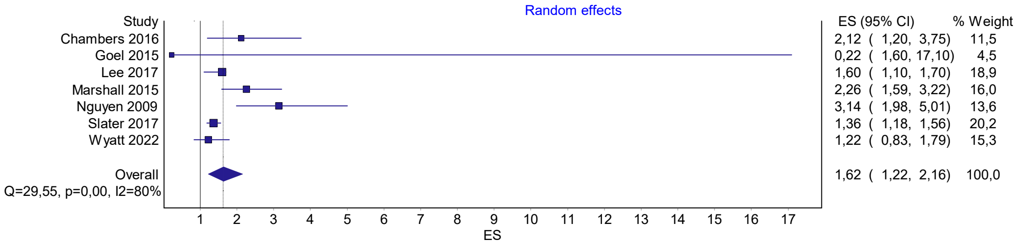

Figure 4F - Pooled effect of navigation services to increase mammography uptake among underserved groups (sensitivity analysis)

Forest plot 4F includes identified studies investigating the effect of navigation services to increase mammography uptake among underserved groups.
In this case, results from Kim 2022, identified as outlier (with a relative weight of 0,1%), and studies judged to be at high risk of bias are excluded.

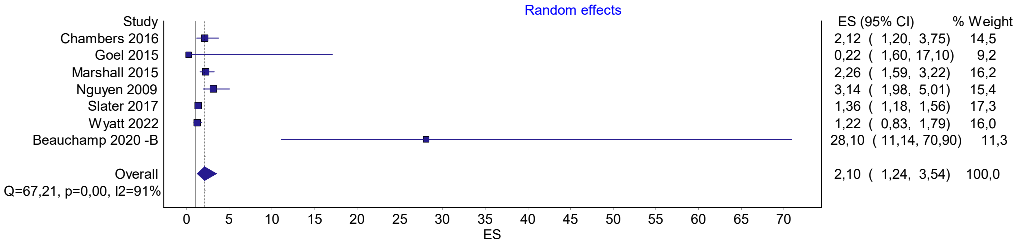

- **Telephonic interventions**

Figure 5A - Overall pooled effect of telephonic interventions to increase mammography uptake

Forest plot 5A includes all identified studies investigating the effect of telephonic interventions to increase mammography uptake.

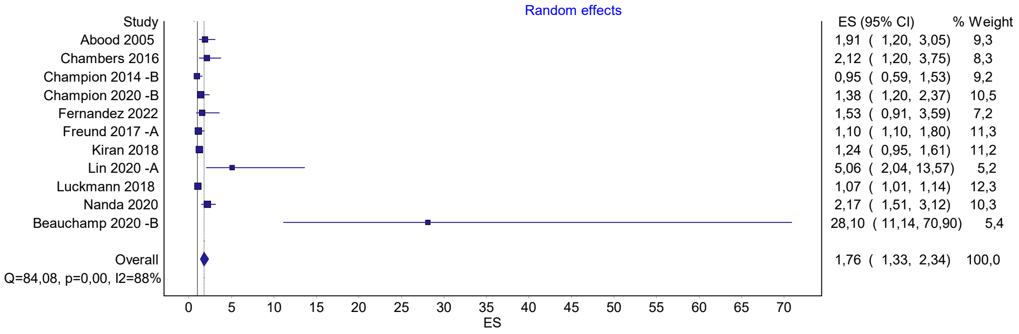

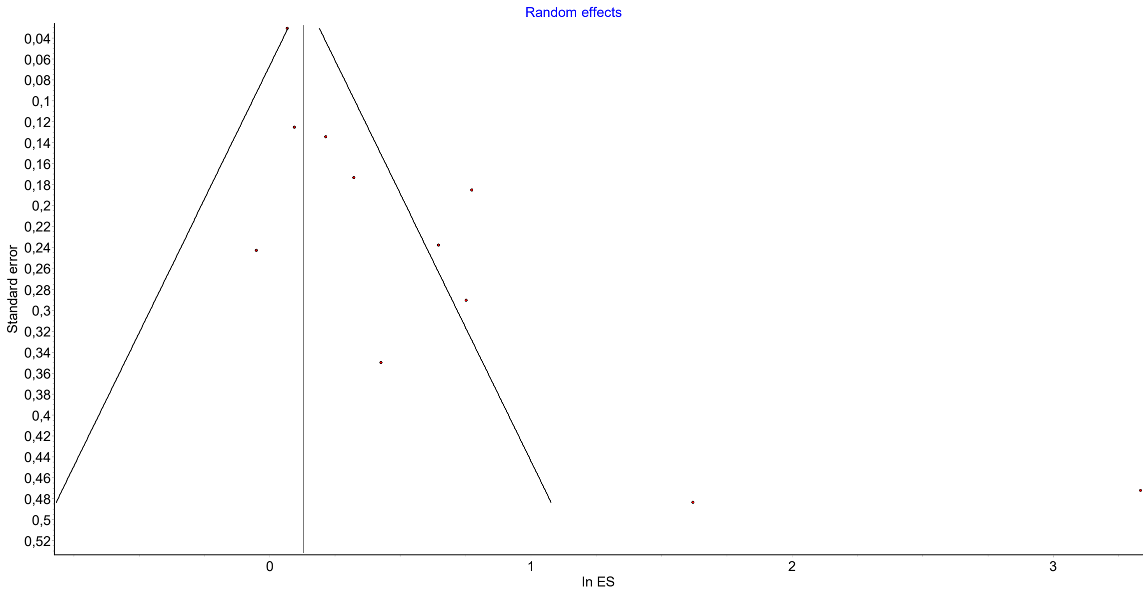

Figure 5B - Overall pooled effect of telephonic interventions to increase mammography uptake (excl. *Beauchamp 2020-B)*

Forest plot 5B includes identified studies investigating the effect of telephonic interventions to increase mammography uptake. In this case, results from Beauchamp 2020-B (with a relative weight of 1%) were excluded from the analysis.

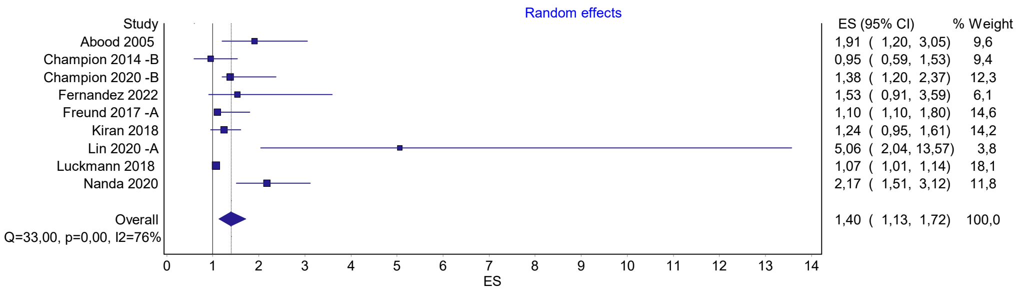

Figure 5C - Overall pooled effect of telephonic interventions to increase mammography uptake (sensitivity analysis)

Forest plot 5C includes identified studies investigating the effect of interventions to increase mammography uptake. In this case, studies judged to be at high risk of bias are excluded.

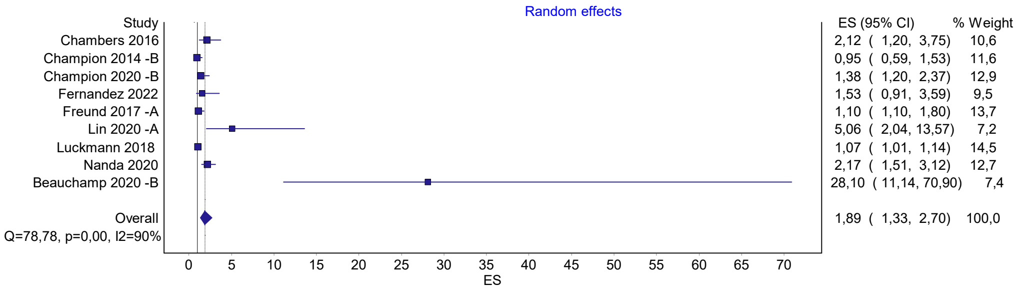

Figure 5D - Pooled effect of telephonic interventions to increase mammography uptake among underserved groups

Forest plot 5D includes all identified studies investigating the effect of telephonic interventions to increase mammography uptake among underserved groups.

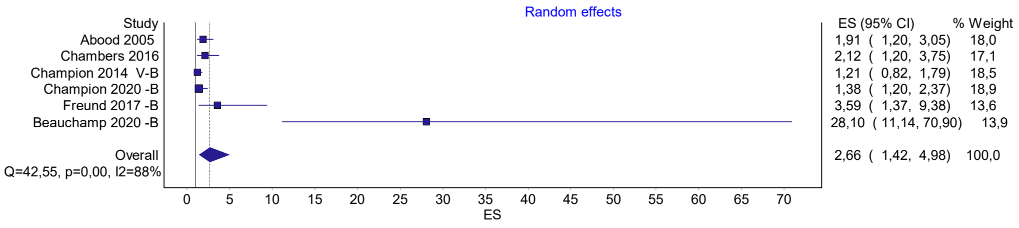

Figure 5E - Pooled effect of telephonic interventions to increase mammography uptake among underserved groups (excl. *Beauchamp 2020-B)*

Forest plot 5E includes identified studies investigating the effect of telephonic interventions to increase mammography uptake among underserved groups.
In this case, results from Beauchamp 2020-B (with a relative weight of 1%) were excluded from the analysis.

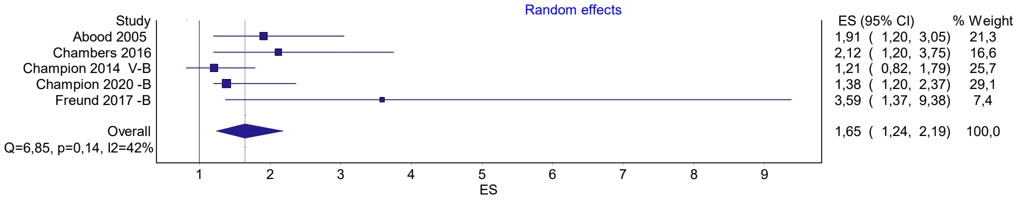

Figure 5F - Pooled effect of telephonic interventions to increase mammography uptake among underserved groups (sensitivity analysis)

Forest plot 5F includes identified studies investigating the effect of telephonic interventions to increase mammography uptake among underserved groups.
In this case, studies judged to be at high risk of bias are excluded.

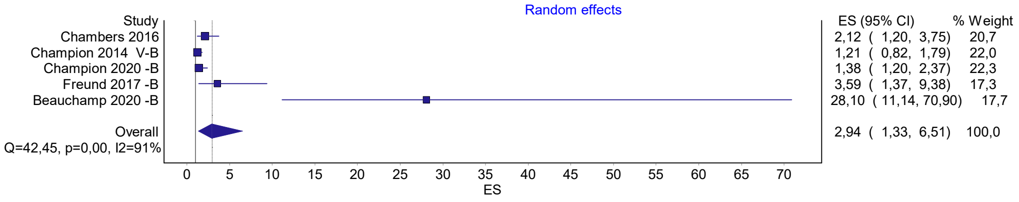

- **<u>Reminders</u>**

Figure 6A - Overall pooled effect of reminders to increase mammography uptake

Forest plot 6A includes all identified studies investigating the effect of reminders to increase mammography uptake.

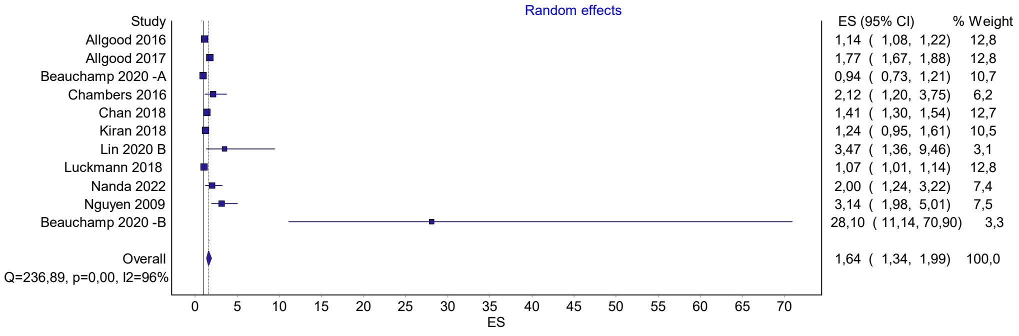

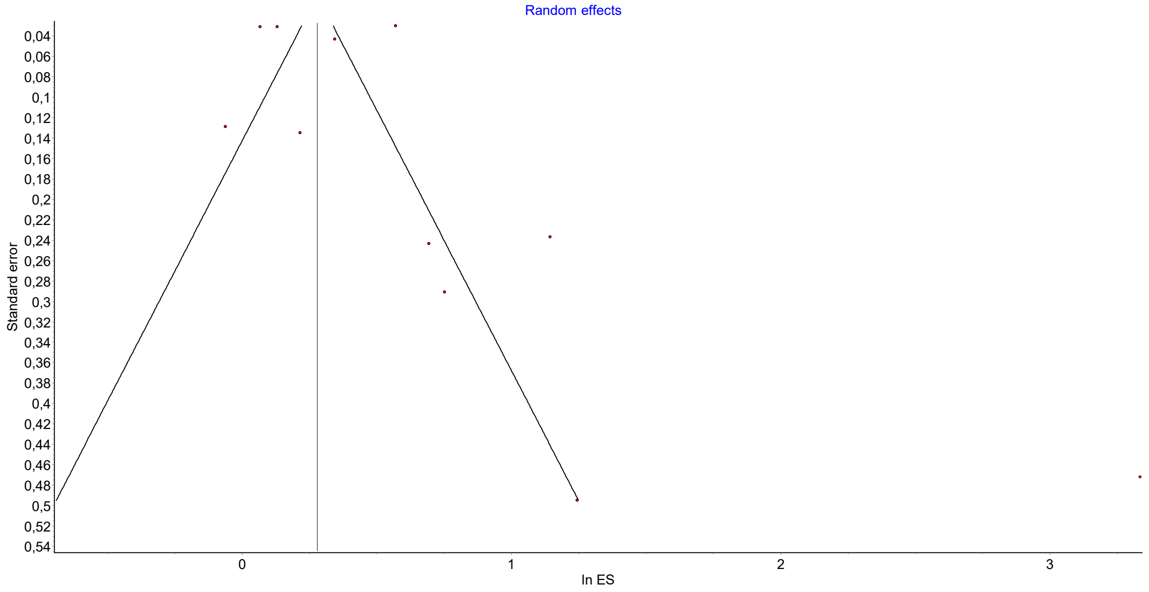

Figure 6B - Overall pooled effect of reminders to increase mammography uptake (excl. *Beauchamp 2020-B)*

Forest plot 6B includes identified studies investigating the effect of reminders to increase mammography uptake. In this case, results from Beauchamp 2020-B (with a relative weight of 1%) were excluded from the analysis.

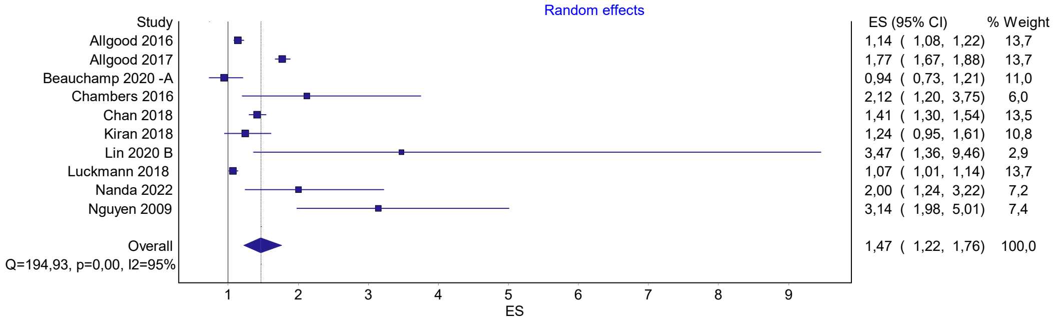

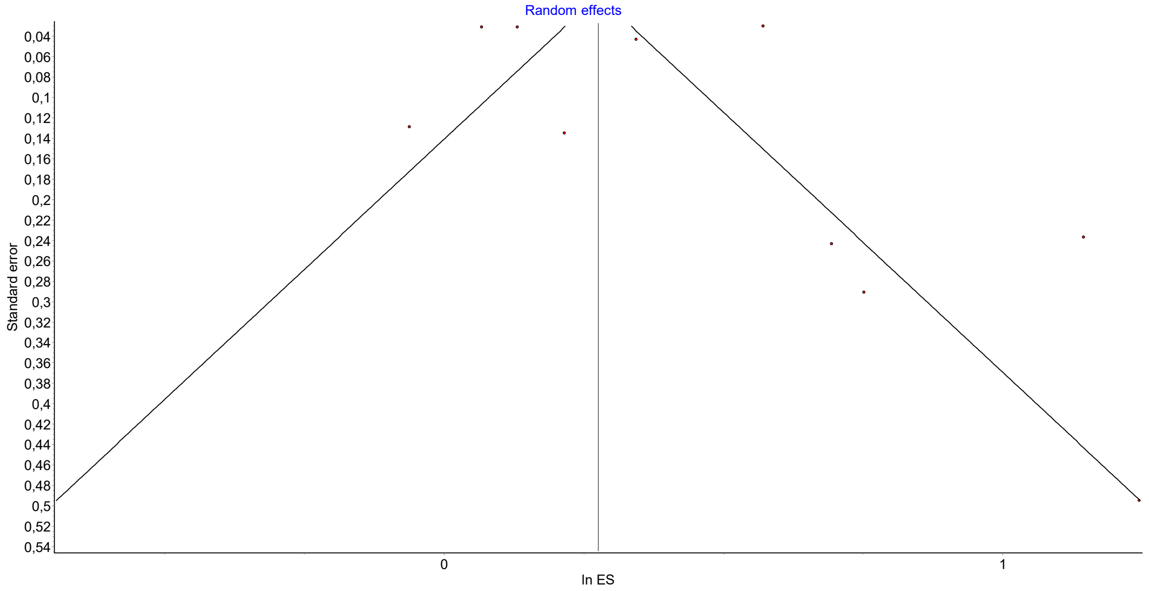

Figure 6C - Pooled effect of reminders to increase mammography uptake among underserved groups

Forest plot 6C includes all identified studies investigating the effect of reminders to increase mammography uptake among underserved groups.

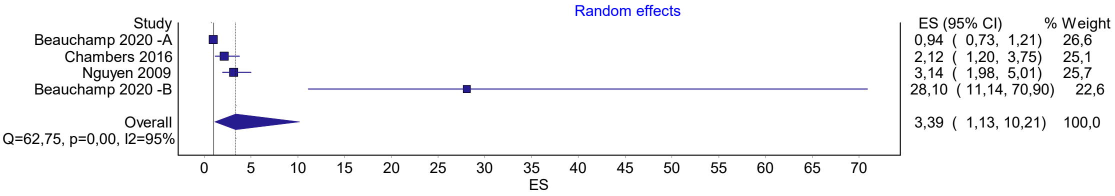

Figure 6D - Pooled effect of reminders to increase mammography uptake among underserved groups (excl. *Beauchamp 2020-B)*

Forest plot 5D includes identified studies investigating the effect of reminders to increase mammography uptake among underserved groups.
In this case, results from Beauchamp 2020-B (with a relative weight of 1%) were excluded from the analysis.

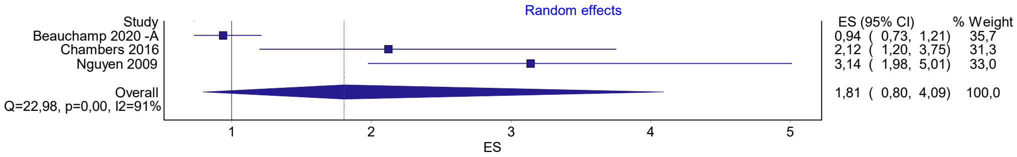

- **<u>Invitation letters</u>**

Figure 7A - Overall pooled effect of invitation letters to increase mammography uptake

Forest plot 7A includes all identified studies investigating the effect of invitation letters to increase mammography uptake.

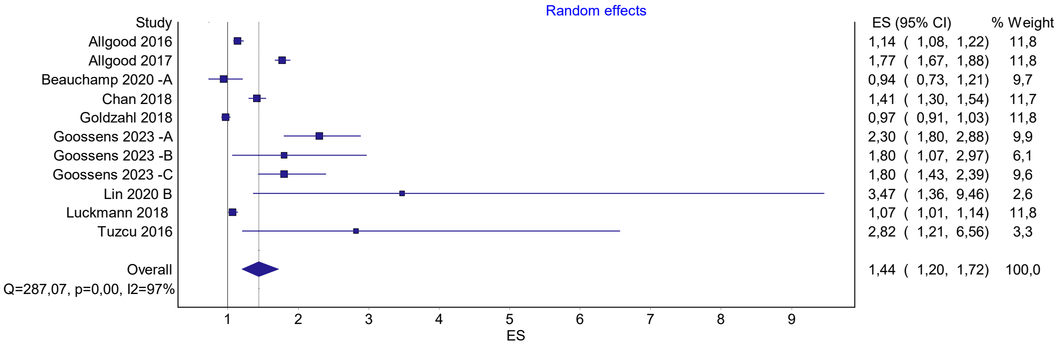

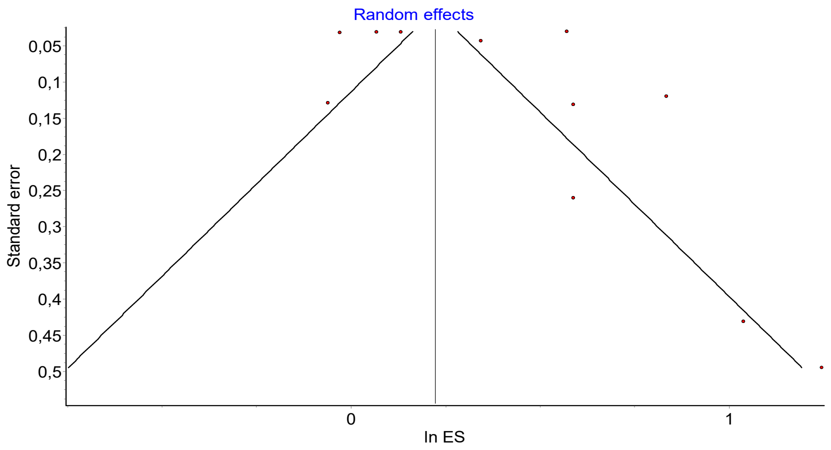

Figure 7B - Overall pooled effect of invitation letters to increase mammography uptake (sensitivity analysis)

Forest plot 7B includes identified studies investigating the effect of invitation letters to increase mammography uptake. In this case, studies judged to be at high risk of bias are excluded.

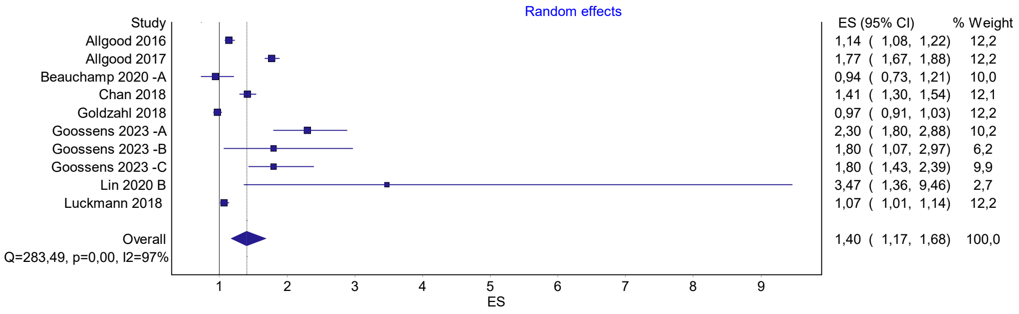

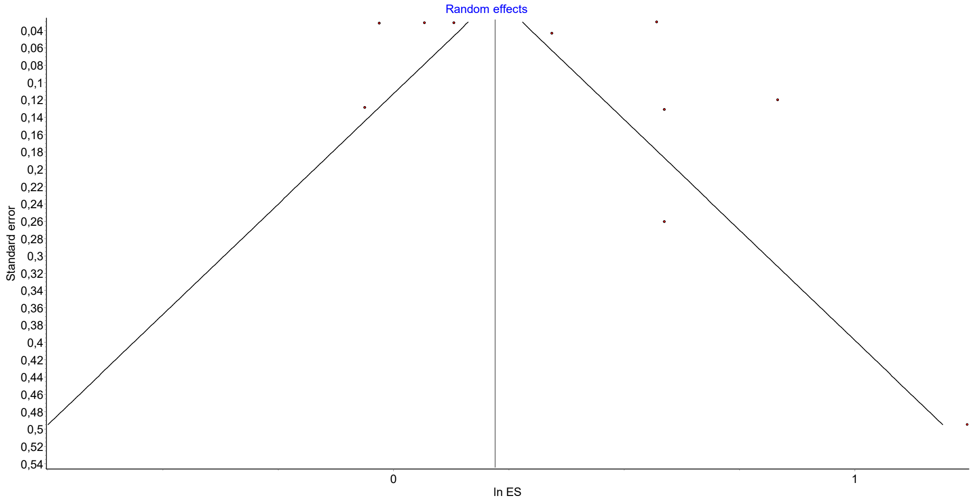

Figure 7C - Pooled effect of invitation letters to increase mammography uptake among underserved groups

Forest plot 7C includes all identified studies investigating the effect of invitation letters to increase mammography uptake among underserved groups.

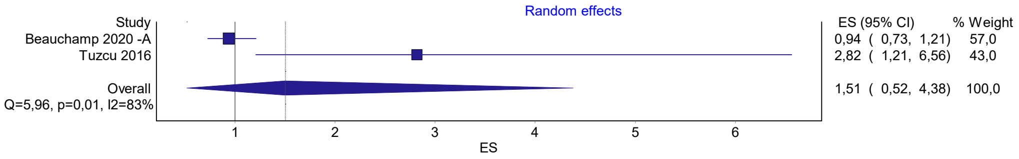

- **<u>Culturally sensitive interventions</u>**

Figure 8A - Overall pooled effect of culturally sensitive interventions to increase mammography uptake

Forest plot 8A includes all identified studies investigating the effect of culturally sensitive interventions to increase mammography uptake among underserved groups.

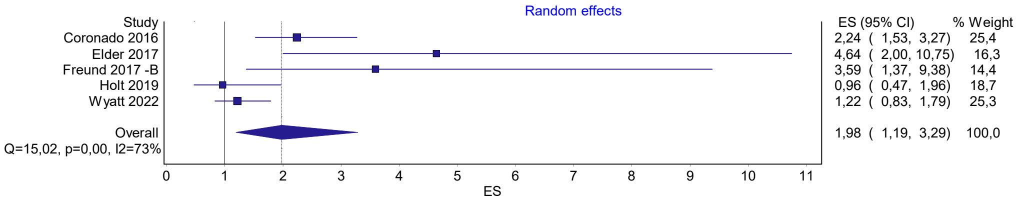

Pooled estimates for studies investigating decision aids, linguistically adapted interventions (eg. materials translated into different languages, using culturally appropriate language or employing communication strategies effective for speakers of a particular language or dialect), smartphone-based interventions (apps / SMS / social media campaigns..), media educational interventions (web, DVD, other platforms..) and printed materials (card, brochure, leaflet, flyer..) were not statistically significant.

- **<u>Linguistically adapted interventions</u>**

Figure 9 - Pooled effect of linguistically adapted interventions to increase mammography uptake among underserved groups

Forest plot 9 includes all identified studies investigating the effect of linguistically adapted interventions to increase mammography uptake among underserved groups.

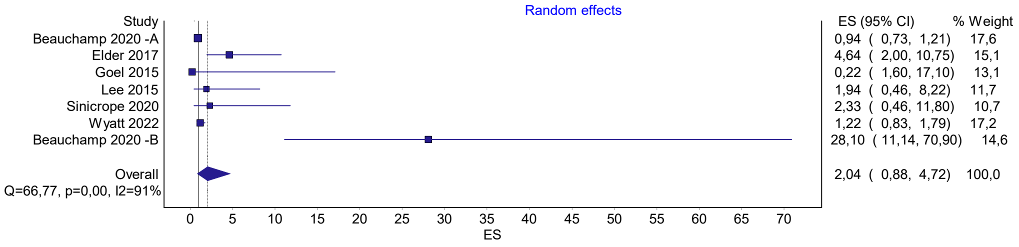

- <u>**Digital-based educational interventions (web, DVD, other platforms..)**</u>

Figure 10 - Overall pooled effect of media educational interventions to increase mammography uptake

Forest plot 10 includes all identified studies investigating the effect of digital-based educational interventions to increase mammography uptake.

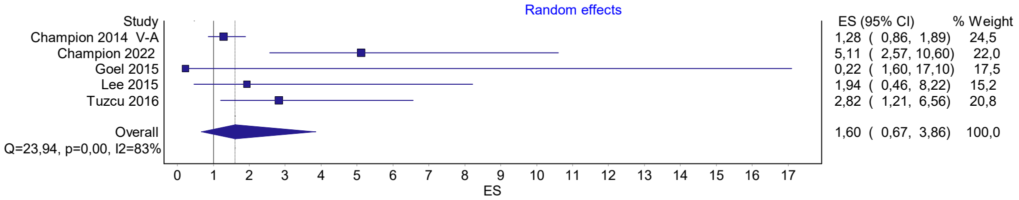

- <u>**Smartphone-based interventions (apps / SMS / social media campaigns..)**</u>

Figure 11 - Overall pooled effect of smartphone-based interventions to increase mammography uptake

Forest plot 11 includes all identified studies investigating the effect of smartphone-based interventions to increase mammography uptake.

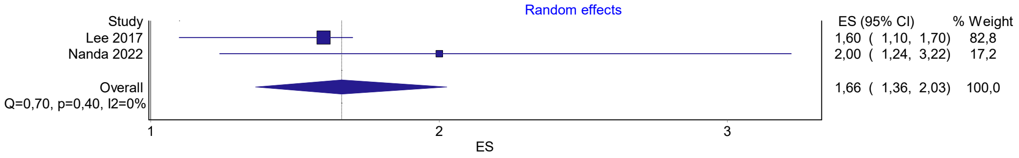

- <u>**Decision aids**</u>

Figure 12 - Overall pooled effect of decision aids to increase mammography uptake

Forest plot 12 includes all identified studies investigating the effect of decision aids to increase mammography uptake.

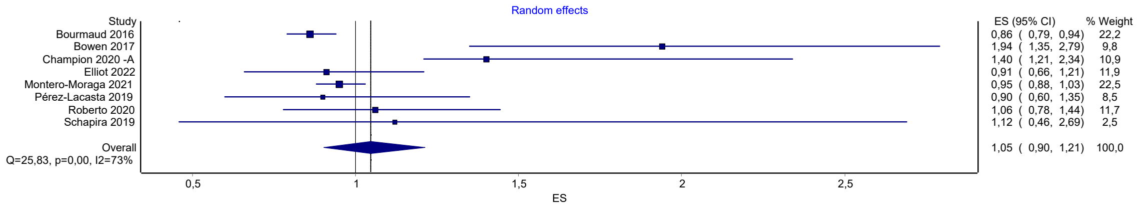

- <u>**Printed material (card, brochure, leaflet, flyer..)**</u>

Figure 13 - Overall pooled effect of printed material to increase mammography uptake

Forest plot 13 includes all identified studies investigating the effect of printed material to increase mammography uptake.

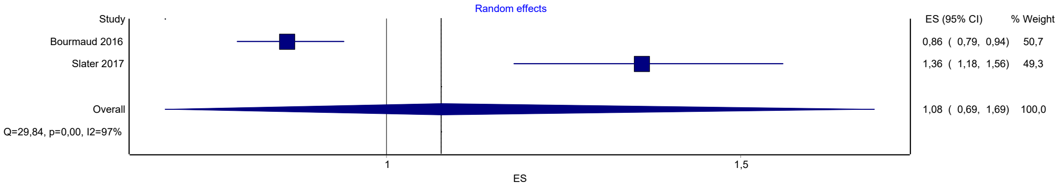

Figure 14 - Risk of bias in randomized studies of interventions (RoB-2) tool

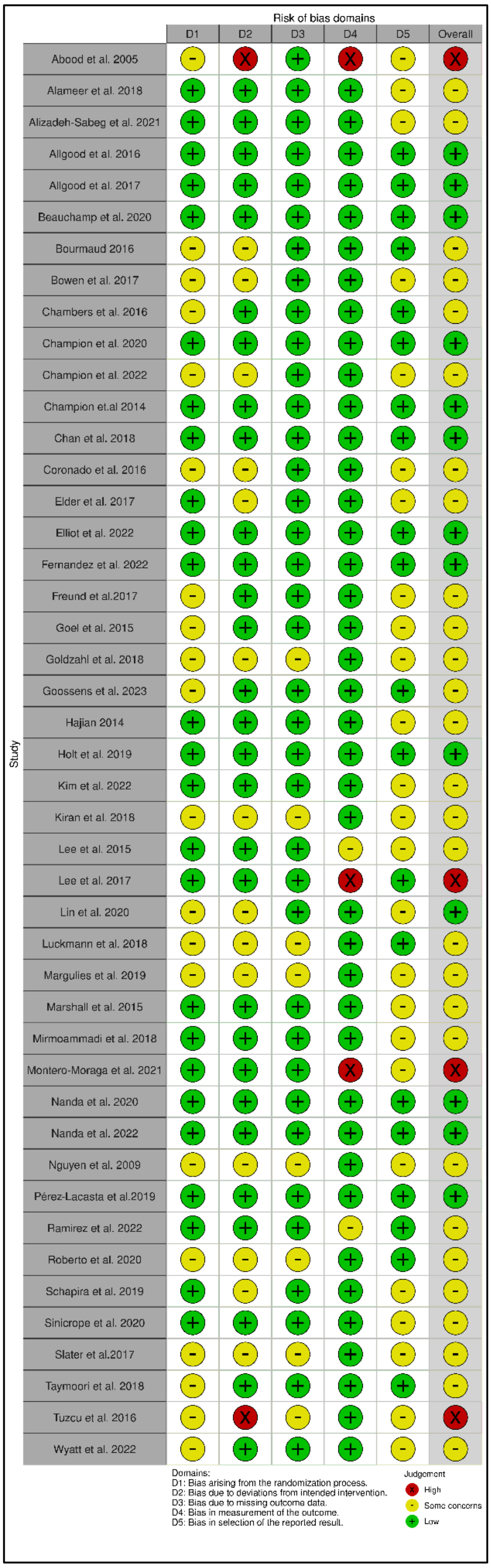

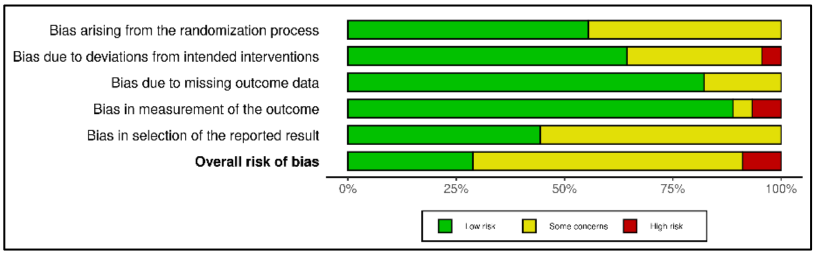

Figure 15 - Risk of bias in non-randomized studies of interventions (ROBINS-I) tool

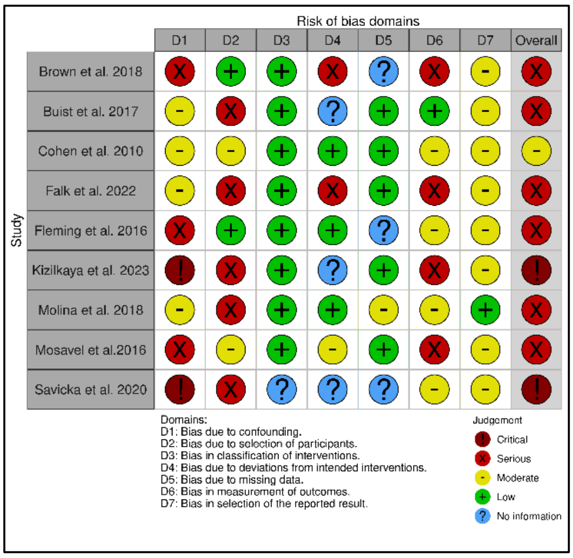

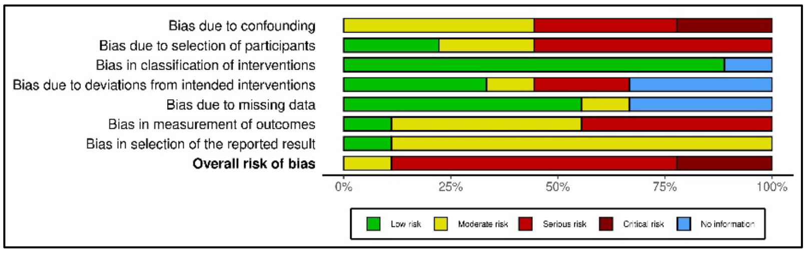

Table 1 - Search strategies

**Generic**	(health AND (promotion OR education OR intervention OR support* OR aid OR aids OR tool OR tools OR instrument OR technolog* OR technique OR techniques OR system OR program* OR process OR method* OR material)) OR ((behavioral OR behavioural) AND (promotion OR education OR intervention OR support* OR aid OR aids OR tool OR tools OR instrument OR technolog* OR technique OR techniques OR system OR program* OR process OR method* OR material)) AND (breast AND cancer AND screening) AND (uptake OR participation OR coverage)
**The Cochrane Library CENTRAL**	Promotion OR education OR intervention OR support* OR aid OR aids OR tool OR tools OR instrument OR technolog* OR technique OR techniques OR system OR program* OR process OR method*OR material in Title Abstract Keyword AND health OR (behavioral OR behavioural) in Title Abstract Keyword AND (breast AND cancer AND screening) AND (uptake OR participation OR coverage) in Title Abstract Keyword - (Word variations have been searched)<br><br>Restrict year range: 2012-2022
**SCOPUS**	(TITLE-ABS-KEY (promotion OR education OR intervention OR support* OR aid OR aids OR tool OR tools OR instrument OR technolog* OR technique OR techniques OR system OR program* OR process OR method* OR material) AND TITLE-ABS-KEY (health OR (behavioral OR behavioural)) AND TITLE-ABS-KEY (breast AND cancer AND screening AND (uptake OR participation OR coverage))) AND (LIMIT-TO (PUBYEAR , 2023) OR LIMIT-TO (PUBYEAR , 2022) OR LIMIT-TO (PUBYEAR , 2021) OR LIMIT-TO (PUBYEAR , 2020) OR LIMIT-TO (PUBYEAR , 2019) OR LIMIT-TO (PUBYEAR , 2018) OR LIMIT-TO (PUBYEAR , 2017) OR LIMIT-TO (PUBYEAR , 2016) OR LIMIT-TO (PUBYEAR , 2015) OR LIMIT-TO (PUBYEAR , 2014) OR LIMIT-TO (PUBYEAR , 2013) OR LIMIT-TO (PUBYEAR , 2012)) AND (LIMIT-TO (LANGUAGE , "English"))
**MEDLINE, Global Health and Biological Abstracts** (via Ovid)	1-17 promotion.ab,kf,ti. OR education.ab,kf,ti. OR intervention.ab,kf,ti. OR support*.ab,kf,ti. OR aid.ab,kf,ti. OR aids.ab,kf,ti. OR tool.ab,kf,ti. OR tools.ab,kf,ti. OR instrument.ab,kf,ti. OR technolog*.ab,kf,ti. OR technique.ab,kf,ti. OR techniques.ab,kf,ti. OR system.ab,kf,ti. OR program*.ab,kf,ti. OR process.ab,kf,ti. OR method*.ab,kf,ti. OR material.ab,kf,ti.<br>18 1 or 2 or 3 or 4 or 5 or 6 or 7 or 8 or 9 or 10 or 11 or 12 or 13 or 14 or 15 or 16 or 17<br>19 health.ab,kf,ti.<br>20 behavioral.ab,kf,ti. OR behavioural.ab,kf,ti.<br>21 18 AND 19<br>22 18 AND 20<br>23 21 OR 22<br>24 breast.ab,kf,ti. AND cancer.ab,kf,ti. AND screening.ab,kf,ti.<br>25 uptake.ab,kf,ti. OR participation.ab,kf,ti. OR coverage.ab,kf,ti.<br>26 24 AND 25<br>27 23 AND 26<br>28 limit 27 to english language

	29 limit 28 to yr="2012 - 2023"<br>30 remove duplicates from 29
**Web of Science**	(((ALL=(promotion OR education OR intervention OR support* OR aid OR aids OR tool OR tools OR instrument OR technolog* OR technique OR techniques OR system OR program* OR process OR method*OR material)) AND ALL=(health OR (behavioral OR behavioural))) AND ALL=((breast AND cancer AND screening))) AND ALL=((uptake OR participation OR coverage))<br><br>Refined By:Languages: English.| Timespan: 2012-01-01 to 2022-12-21 (Publication Date)
**Google scholar**	Health|(behavioral|behavioural) (promotion|education|intervention|support|aid|tool|instrument|technolog|technique|system|program|process|method|material) breast cancer screening (uptake|participation|coverage)<br><br>Restrict year range: 2012-2022

Table 2 – GRADE Summary findings on the effectiveness of interventions to increase breast cancer screening

Outcome	Number of participants<br>Number of studies	Relative effect or Difference<br>(95% CI)	Certainty	Reasons for judgement
**Mammography uptake**<br>Overall	161,141<br>44 RCTs	OR 1.50<br>(95%CI 1.36-1.67)<br>P < 0.001	⊕⊕⊕<br>**MODERATE**	• Risk of bias: No downgrade<br>Most studies have low or unclear risk of bias and - as demonstrated by sensitivity analysis - the potential limitations are not likely to lower confidence in effect.<br>• Imprecision: No downgrade<br>95%CI exclude a OR of 1.00 and the total number of events exceeds the IOS criterion.<br>• Inconsistency: Downgrade -1<br>Serious inconsistencies due to unexplained heterogeneity.<br>• Indirectness: No downgrade<br>The review question was addressed according to the predefined eligibility criteria and PICO.<br>• Publication bias: No downgrade<br>Asymmetrical funnel plot reveal risk for publication bias, however, a systematic search was conducted including trial databases and sources of grey literature.
**Mammography uptake**<br>Underserved groups	14,720<br>19 RCTs	OR 1.63<br>(95%CI 1.34-1.97)<br>P < 0.001	⊕⊕⊕<br>**MODERATE**	• Risk of bias: No downgrade<br>Most studies have low or unclear risk of bias and - as demonstrated by sensitivity analysis - the potential limitations are not likely to lower confidence in effect.<br>• Imprecision: No downgrade<br>95%CI exclude a OR of 1.00 and the total number of events exceeds the IOS criterion.<br>• Inconsistency: Downgrade -1<br>Serious inconsistencies due to unexplained heterogeneity.

- Indirectness: No downgrade
  The review question was addressed according to the predefined eligibility criteria and PICO.
- Publication bias: No downgrade
  Asymmetrical funnel plot reveal risk for publication bias, however, a systematic search was conducted including trial databases and sources of grey literature.

***The risk in the intervention group** (and its 95% confidence interval) is based on the assumed risk in the comparison group and the relative effect of the intervention (and its 95% CI)

CI: confidence interval; OR: odds ratio; OIS: optimal information size;

Domains assessed included risk of bias, imprecision, inconsistency of results, indirectness of evidence, and publication bias

GRADE Working Group grades of evidence

⊕⊕⊕⊕ **High certainty:** We are very confident that the true effect lies close to that of the estimate of the effect

⊕⊕⊕ **Moderate certainty:** We are moderately confidence in the effect estimate: The true effect is likely to be close to the estimate of the effect, but there is a possibility that it is substantially different

⊕⊕ **Low certainty:** Our confidence in the effect estimate is limited: The true effect may be substantially different from the estimate of the effect

⊕ **Very low certainty:** We have very little confidence in the effect estimate: The true effect is likely to be substantially different from the estimate of effect

Table 3 – Description of interventions and comparators

Author / year	Intervention type	Intervention description	Conceptual framework	Comparator	Comparator description
Abood et al. 2005	Telephonic intervention	In this loss-framed approach, a client/patient is faced with a risky decision (risking the detection of a malignancy through mammography), but this message is combined with information about the high efficacy of the desired action (mammography). This makes individuals more likely to avoid a potential loss (a late-stage diagnosis) by taking action (obtain a mammogram).	Loss-framed approach	Standard clinic telephone message	Obtaining eligibility information and the offer of a mammogram appointment
Alameer et al. 2018	Educational intervention	HBM-based standardized health education program (SHEP) developed by the Heath Education Committee of the Health Education and Promotion Department, Faculty of Public Health and Tropical Medicine, Jazan University (Supplementary Material 1). SHEP included a comprehensive lecture about BC, with a deep focus on detection and screening tools, illustrated with a PowerPoint presentation containing pictures and videos. The program also included a practical BSE session. Each educational session lasted 60 min. At the end of SHEP, a focused group discussion was conducted to answer participants' questions, and to discuss important barriers regarding BSE practice and visiting primary health care centers or clinics to undergo CBE and mammography. Several scientific and administrative solutions were discussed, with a concentration on the benefits of screening tools, both to overcome those barriers and to motivate the participants to utilize breast cancer screening tools. The interventions were conducted by three health care assistants who were trained by the Health Education Committee and the primary investigator.	Health belief model	Pamphlets	Included general information about BC, without any interaction with our defined measured variables.
Alizadeh-Sabeg et al. 2021	Educational intervention	The intervention group participated in six sessions (two educational and four MI sessions) in groups of 8–12 women. These 60−90 min sessions were held weekly. All counseling sessions were conducted by the first author who was a trained and certified counselor in MI. The two educational sessions included the following discussions:	Motivational interviewing, Trans-theoretical model	Standard care	NA
Allgood et al. 2016	Invitation letter, Reminder	An invitation reminder was sent 2 weeks after the initial invitation letter, reminding them of their fixed appointment. Participants received this letter approximately 7 days before their original screening appointment.	NA	No intervention	NA
Allgood et al. 2017	Invitation letter, Reminder	The intervention consisted of an invitation to a second appointment with fixed date and time.	NA	No intervention	NA
Beauchamp et al. 2020 (A)	Invitation letter, Linguistically adapted intervention, Reminder	A routine reminder letter in the preferred language spoken at home (the text was slightly altered from the English letter and contained brief, simplified information about the purpose of screening, information about how to book a screen, and contact details for BSV. In addition, a photo and quote from an Italian or Arabic GP were included). The original invitation in English was also provided.	NA	Standard care	Reminder letter in English (containing information about booking, statistics ect..)
Beauchamp et al. 2020 (B)	Telephonic intervention, Navigation, Linguistically adapted intervention, Reminder	Telephone call (4-8 minutes) in the preferred language to remind and assist them to book a screening appointment. The script included an introduction by the caller and the purpose of the call. The caller was able to make a booking during the phone call, and advice provided about where attend for screening. Callers could deviate from the script if required.	NA	Standard care	Reminder letter in English (containing information about booking, statistics etc..)
Bourmaud et al. 2016	Decision aid, Printed materials (card, brochure, leaflet, flyer..)	A printed decision aid, known as the DECIDEO leaflet, was developed following international guidelines for the 'provision of information and the construction of decision aid tools. This tool is a 12-page pocket leaflet presenting the different health decision options and providing probabilities of the outcomes according to the choices made, highlighted by illustrations and histograms. Available at supplementary materials: https://www.ncbi.nlm.nih.gov/pmc/articles/PMC4914328/	NA	Standard care	Regular invitation letter (with set time and date)

Bowen et al. 2017	Decision aid	A specialized Web site contained and delivered all informational materials pertaining to breast cancer, genetic risk, and associated issues and links. The content was based our successful counseling and tailored print message interventions that increased mammography and quality of life in women in the same age group as was recruited for this study. For each participant it was included an online personal risk page, based on the Gail score.	Self-regulation model of health behavior	Standard care	Regular invitation letter (with set time and date)
Brown et al. 2018	Health consultation, Navigation, Economic support/Voucher	20- to 45-minute health screening that included referrals to clinical and community resources and 15- to 30-minute telephone follow-up interviews at 1 month, 3 months, and 6 months after the initial screening. Uninsured, underinsured, and Medicaid-eligible participants could receive vouchers for breast, cervical, and colorectal cancer screening.	NA	Before	NA
Chambers et al. 2016	Telephonic intervention, Navigation, Reminder	Within two weeks of the er letter being posted, the research group made a simple telephone call to them that they had not attended their scheduled appointment and to provide information on how they could rearrange this appointment; People were asked to describe any reasons they had for missing their scheduled appointment, and where appropriate, any barriers they mentioned were addressed using a pre-specified list of responses, generated from previous research (data available from the authors). Any patient queries or concerns about the breast screening appointment were also addressed, using responses from existing screening materials. Participants were then asked whether they intended to make an appointment to attend for breast screening, whether they minded being telephoned about breast screening, and whether they found the telephone call helpful in addressing any concerns/queries they may have had. There were also two questions relating to Anticipated Regret.	NA	Standard care	Regular invitation letter (with set time and date)
Champion et al. 2014 (A and V-A)	Digital-based educational intervention (web, DVD, other platforms..)	The tailored interactive DVD had a function for which participants could respond to questions using the arrows on the remote. The DVD began with a narrator introducing the program. Four women representing different demographic profiles were selected to deliver intervention messages. An animation of breast cancer developing and metastasizing was shown. Women who had not had a previous mammogram viewed a video of the mammography process. Women were queried about beliefs, fataslism and barriers that would prevent them from receiving a mammogram. Massages to address these barriers were provided throughout the program. The DVD program ended with the narrator encouraging viewers to make a mammography appointment.	Health Belief Model and Transtheoretical Model	Standard care	NA
Champion et al. 2014 (B and V-B)	Telephonic intervention	The telephone intervention was developed using the same tailoring variables and messages used in the interactive DVD (Champion et al. 2014 A and V-A).	Health Belief Model and Transtheoretical Model	Standard care	NA
Champion et al. 2020 (A)	Decision aid	The web-based program providing tailored messages based on the individual's knowledge; perceived and actual risk of cancer; and benefits, barriers, and self-efficacy for screening. After women entered answers to queries, tailored messages were delivered in real time through an algorithm built into the interactive program. Video clips illustrated the screening procedures of mammography. Audio dialogue accompanied each question, allowing women with low literacy to use the program. Women who had not completed the intervention by 4 weeks were sent a reminder e-mail.	Theory of Planned Behavior, Health Belief Model, and Transtheoretical Model	Standard care	NA
Champion et al. 2020 (B)	Telephonic intervention	A phone-based tailored intervention (average time of 19 minutes) delivered tailored messages consistent with algorithms used in the web-based programming. Participants were asked if they wanted a mailed scheduling an appointment. Phone interventionists were trained during a 2-day program with role playing.	Theory of Planned Behavior, Health Belief Model, and Transtheoretical Model	Standard care	NA
Champion et al. 2022	Digital-based educational intervention	An interactive, tailored DVD was developed to provide information for any cancer screening-breast, cervical or CRC-for which participants were not UTD. Participants who were not UTD	NA	Standard care	NA

	(web, DVD, other platforms..), Navigation	with breast cancer screening guidelines were directed to view the tailored mammography screening program included in the DVD. The DVD was based on a previous efficacious technology-based screening intervention developed by study investigators. Participants used a DVD remote control to answer questions presented on menus within the DVD, and a tailored algorithm provided appropriate messages based on individual inputted responses. Content for interactive messages within the DVD were supported by a theoretical framework and were revised from an extensive message library used in prior research. Tailored messages were delivered based on each user's cancer screening history; knowledge of and risk factors for breast cancer; perceived benefits and barriers to breast cancer screening; and self-efficacy to obtain breast cancer screening. Information about scheduling and completing breast cancer screening was also included in the DVD.			
Chan et al. 2018	Invitation letter, Reminder	Reminder letter signed by family physician (drafted based on feedback from focus groups evaluating prior written communication materials and then edited in consultation with members of the BC Cancer Agency's Family Practice Oncology Network). A regular reminder postcard was included.	NA	Standard care	Regular reminder postcard
Cohen et al. 2010	Telephonic intervention, Culturally sensitive intervention	Phone call performed by a social worker with nursing education. Based on 5 focus groups previously conducted with Arab women, a list of scripted answers to barriers and another list of religious and cultural promoters were prepared to address each of the 5 areas of cultural barriers (exposure of body, social barriers, religious beliefs about cancer and health, environmental barriers, uneasiness with own body). For example, regarding an answer to the traditional barrier of perceiving cancer as an immutable fate, the interviewer stressed the view (rooted in Muslim and Christian writings) of the woman's responsibility for her own health and presented facts on survival rates in cases of early detection; the social barrier of fear of being seen at the clinic was answered by stressing the change in Arab society and the growing understanding of the importance of prevention. Scripted responses were also created for general barriers, such as fear of harm to the body or of pain. For example, fear of pain was answered by pointing out that the pain experience lasted for only a few seconds and then disappeared, teaching the woman distraction techniques, and suggesting scheduling mammography a week after the end of the menstrual period (this technique also increases sense of control). To create a sense of obligation, at the end of the session, the woman was asked about her intentions regarding breast examination.	Transtheoretical model, Health belief model, Cultural competence approach	Standard care	NA
Coronado et al. 2016	Educational intervention, Culturally sensitive intervention	Promotoras are lay community members who receive specialized training to deliver health education in the community. Each patient randomized to the intervention received a home visit from a promotora, who engaged her in a discussion about breast cancer prevention. The promotora followed principles of motivational interviewing, a patient-centered counseling approach that is considered culturally responsive because counselors can incorporate issues related to social context into the discussion. Two weeks after the home visit, the promotora made a follow-up telephone call to the woman to review any planned action steps and assess readiness to schedule a mammogram.	NA	Standard care	NA
Elder et al. 2017	Educational intervention, Culturally sensitive intervention, Linguistically adapted intervention	NA	NA	Physical activity promotion	NA
Elliot et al. 2022	Decision aid	A cancer prevention algorithm (Web-based Clinical Decision Support CDS) based on USPSTF was linked with electronic health records of patients in order to produce personalized output recommendations to both PCPs and patients in both printed and electronic formats. All recommendations were presented as suggestions, and the interface emphasized CDS suggestions do not take the place of clinical judgment or override a PCP's detailed knowledge	NA	Standard care	NA

		of a patient. The shared-decision making tool included notification for when a patient was due for screening and a brief presentation about benefits and risks, options to consider, and invitation to access the full-length SDM tool during the clinic visit and discuss with their provider. The full-length SDM tool was long 3-4 pages and advised the patient and PCP to conduct cancer screening as recommended by the USPSTF.			
Falk et al. 2022 (A)	Navigation	Women received a form (help request) to indicate their service needs and barriers to screening. For example, a woman was told by a local primary care physician of the program payment assistance to receive a mammogram. Rather than wait for an education event, she could immediately request PN services to get connected with a provider and receive a payment voucher. In this case, the participant would not complete a pre- and posttest as she did not attend the education event but still received PN services.	NA	Standard care	NA
Falk et al. 2022 (B)	Educational intervention, Navigation	Women attended a “pink party” that educated them about cancer screening, shared a breast or cervical cancer survivor’s story about their cancer journey, and included a presentation by a medical professional (oncologist, nurse, radiologist, etc.), who answered questions from the women regarding screening. These events usually occurred once per program year and rotated within each region based on the capacity of the local navigators and the requests of the counties in the region. To request navigation, participants at the “pink party” completed a simple paper form (help request) with their me and telephone number at the end of the event requesting help with screening.	NA	Standard care	NA
Fernandez et al. 2022	Telephonic intervention, Navigation	The navigators called participants within one working day of participant assignment to the R&N condition. These calls included building a collaborative relationship with callers, identifying their needs, working with them to identify barriers to services and coordinate solutions, and providing logistical (e.g., making appointments) and emotional support.	Social Cognitive Theory	Standard telephonic referral	Referrals specific to their cancer control needs (i.e., me and contact of service provider).
Fleming et al. 2016	Invitation letter	The intervention arm implemented an alternative process for inviting women, in which they were sent a letter inviting them to phone their screening unit and make an appointment at a date and time convenient to them.	NA	Standard care	Regular invitation letter
Freund et al. 2017 (A)	Telephonic intervention, Culturally sensitive intervention	Telephonic interview, performed by a social worker with nursing education, for which a list of scripted answers to barriers and a list of religious and cultural promoters were prepared to address 5 areas of cultural barriers (exposure of body, social barriers, religious beliefs about cancer and health, environmental barriers, uneasiness with own body).	Culture-Based Health Belief	No intervention	NA
Freund et al. 2017 (B)	Telephonic intervention, Culturally sensitive intervention	Telephonic interview, performed by a social worker with nursing education, for which a list of scripted answers to barriers and a list of religious and cultural promoters were prepared to address 5 areas of cultural barriers (exposure of body, social barriers, religious beliefs about cancer and health, environmental barriers, uneasiness with own body).	Culture-Based Health Belief	No intervention	NA
Goel et al. 2015	Digital-based educational intervention (web, DVD, other platforms..), Navigation, Linguistically adapted intervention	The women viewed the video using a PC laptop computer equipped with Windows Media Player and an individual headset in the waiting area of the clinic. The video demonstrates both of these concepts through a brief, thirty second encounter between a patient and provider. In this demonstration, the provider sets a chronic disease-focused agenda for the visit, while the patient acknowledges the chronic disease agenda and prompts the provider about her mammogram referral. This portion demonstrates the language a patient can use to engage the provider and aims to increase the viewer's skill and comfort level in requesting a recommended referral (vicarious learning). The provider acknowledges the patient’s request and states she will see if the patient is due (positive outcome expectancy).In addition to the novel features described, the video explains the importance of mammography and demonstrates the experience of undergoing mammography. This video can be viewed at the Northwestern University General Internal Medicine website http://cch.northwestern.edu/edtools/.	Social Cognitive Theory	Standard care	An electronic health record system provides clinical decision support to health care providers, including an alert system for overdue preventive health services such as mammography. This system was in effect and unchanged throughout the duration of the study. In addition, all sites have preventive health “navigators” that track individual patients who have already received mammogram referrals and provide assistance in addressing attitudil or logistic barriers to mammography. All women in the study, both intervention and control, had access to these services.

Goldzahl et al. 2018	Invitation letter	The official logos of the three National Health Insurance funds provided a clear signal that the letters came from an official institution. The official letter from the National Institute of Cancer provided precise information regarding the magnitude of the risk, starting with the sentence: "Over the course of her life, nearly one in eight women will face this cancer".	Social norms framework	Standard care	Regular invitation letter
Goossens et al. 2023 (A - B - C)	Invitation letter	A letter with a prescheduled appointment (this is the standard practice in the PMSP).	NA	Open invitation letter	A letter which explained the letter served as an open invitation to screening and asked them to contact the screening unit or call a toll-free number for an appointment.
Hajian et al. 2011	Educational intervention	HBM constructs based intervention (well-known psychological theories health education for breast cancer screening)	Health belief model	Standard care	NA
Holt et al. 2019	Educational intervention, Culturally sensitive intervention	Education via church community health advisors (CHAs) trained with a web training module (with a self-paced online system where to log in and out). Once certified by passing a knowledge examination that included items on sex-specific cancers (e.g., only the women received breast cancer knowledge items). After certification, using Project HEAL intervention materials (e.g., recruitment materials, PowerPoint presentations) they implemented a series of three monthly educational workshops in their churches on cancer early detection.	NA	Standard care	NA
Kim et al. 2022	Educational intervention, Navigation	Cancer prevention program and telephone counseling provided by trained CHW	NA	Standard care	NA
Kiran et al. 2018	Telephonic intervention, Reminder	NA	NA	Reminder letter	NA
Kizilkaya et al. 2023	Educational intervention, Navigation	A physician conducted an interactive BC education session with education materials written by the authors of the study, also verbally via telephone, with each participant. The education session contained a comprehensive review of breast cancer etiology, risk factors, clinical manifestations, screening, diagnosis, treatment, and prevention. The education intervention was administered with the assistance of a female certified interpreter, approximately 1 h in duration. Social work services, including assistance with appointment scheduling and transportation to the healthcare facility, were offered to each patient to assist with completion of screening mammography.	NA	Before	NA
Lee et al. 2015	Digital-based educational intervention (web, DVD, other platforms..), Family member intervention, Linguistically adapted intervention	Web-based KIM-CHI program, including (a) two part video showing a 30-min Korean-language film, (b) PowerPoint summary of the study (c) homework for each couple (wife and husband) and requiring to then call the study phone number to leave a message about what they discussed.	NA	Standard care	NA
Lee et al. 2017	Smartphone-based interventions (apps / SMS / social media campaigns..), Navigation	mMammogram mobile phone app, combined with health navigator services	Health belief model	Brochure	NA
Lin et al. 2020 (A)	Telephonic intervention, Navigation	The participants were phoned by public healthcare providers and given the same information as the mail reminder group. Participants in the telephone group (TR group) could be assisted by health care providers to schedule a mammography screening if needed.	NA	Standard care	The usual care provided via postcard with free mammography screening information was given to the control group by the public healthcare center within 1 month after they had turned 45 years of age.
Lin et al. 2020 (B)	Invitation letter, Reminder	The mailed information was checked by the experts on public health and breast cancer to ensure that it was adequate and proper.	NA	Standard care	The usual care provided via postcard with free mammography screening information was given to the control group by the public healthcare center

					within 1 month after they had turned 45 years of age.
Luckmann et al. 2018	Invitation letter, Telephonic intervention, Navigation, Reminder	Women were called 1-2 weeks after a reminder letter was sent if no mammogram was scheduled. The caller confirmed that the patient was due for a mammogram and offered to schedule one. Up to five call attempts were made at different times with two voicemail messages requesting a call back "about a test that is coming due."	NA	Standard care	Invitation and reminder letter
Margulies et al. 2019	Health consultation, Navigation	After contact with a primary care physician, first-year medical students trained by the principle investigator performed motivational interviewing (Rollnick et al.) about breast cancer and mammograms, existing barriers to getting mammograms and way to overcome these, and were able to escort women to the radiology suite for a walk-in mammogram appointment.	NA	Standard care	NA
Marshall et al. 2015	Navigation	Navigators made introductory phone calls to introduce their role, review baseline screening status, discuss printed educational materials, consider predisposing factors to cancer screening (e.g., perceptions and beliefs about cancer), and identify potential barriers. Navigators helped arrange appointments and accompany participants to screenings when necessary. Navigators also worked to enhance the patient-provider interaction by coaching patients on potential questions to ask their providers. Over the course of the study, the majority of patient navigators were African American (71 %).	NA	Printed Educational Materials (PEM)	Printed Educational Materials (PEM) containing general information about cancer and preventive services covered by Medicare.
Mirmoammadi et al. 2018	Educational intervention	90 minutes group consultation held by Midwifery consultants with 10 years' experience, in which the use of two techniques was combined. The GATHER technique includes G (Greeting) as respect to the client; A (Ask) as asking from the volunteer regarding the knowledge, attitude and the reason of attending the consultation and helping the volunteer to express her demands, beliefs and emotions, T (Tell) the answer to the clients questions, H (Help) helping the volunteer to make an appropriate decision, E (Explain) as explaining the matters that are necessary for reaching the aim and R (Return) follow-up sessions or meeting after intervention in intervention centers (Rinehart et al., 1998). HBM constructs training was conducted according to women perceived susceptibility, severity, self-confidence, health motivation, benefits and barriers of breast cancer screening. Training booklet were handed out, consisting of information about breast atomy, physiological changes in the breast, symptoms and signs of breast cancer, methods of breast cancer screening and high treatment rates of breast cancer.	Health Belief Model, GATHER consultancy technique	Standard care	NA
Molina et al. 2018	Community intervention, Epowerment intervention, Navigation,	Contextualizing breast health via women's informal social networks (interpersonal advocacy) and larger communities (community advocacy). The empowerment intervention consists of a session focused on breast cancer and methods of early detection, a session focused on one-on-one conversations with family and friends about breast cancer, and a session focused on health-related volunteerism.	Social cognitive theory, Volunteerism, Cognitive dissonance theory	Education Program	Community health workers testimonial as a survivors and family members/caregivers of survivors; Group discussions related to breast health; Individual activities (Identification of risk factors; Economic, psychosocial, logistic barriers; Preferred solutions/plans for screening); Educational interventions of which: a session focused on breast cancer and methods of early detection, a session focused on diet as a method of prevention, and a session focused on physical activity as a method of prevention
Montero-Moraga et al. 2021	Decision aid	The intervention consisted of adding an information leaflet to the letter. This leaflet, signed by the Catalonian Health Department, describes the breast cancer screening program, and how it is implemented in the region. It explains that in most cases the screening program is beneficial but that it also has risks, and therefore women make the fil decision on participation. The leaflet contains qualitative and quantitative information on breast cancer, mortality reduction due to mammography screening, the possibility and advantages of detecting early-stage cancer in participants, and explains how the mammogram is performed.	NA	No intervention	NA

		The leaflet also gives the absolute number of breast cancers diagnosed in the region and the number of women who die as a consequence. It also provides the number of women who will need to undergo additional diagnostic tests per 1000 women who participate, the number of cancer diagnoses among them and how many deaths are avoided per 1000 women who regularly attend screening every 2 years from the ages of 50 to 69 years. The leaflet also provides information on the potential risks of screening, explains the meaning of overdiagnosis and overtreatment, and false positives and false negatives. Furthermore, it provides numerical estimates of overdiagnosis, expressed as absolute risk per 1000 participants.			
Mosavel et al.2016	Educational intervention, Family member mediation	A trained facilitator provided to the Youth a 15-min PowerPoint presentation to groups of four to six participants. She introduced cancer disparity data specific to African Americans as well as the American Cancer Society screening recommendations. After the PowerPoint, Youth were provided with a sealed envelope that contained specific information about the screening that their mother or adult relative needed. The youth had the opportunity to role play and practice delivery of a personalized screening message to their relative with the group facilitator. Youth were asked to text the project coordinator once they delivered the message to their relative. They also received a pamphlet titled “The 3Gs of cancer screening. What your relative should know. What YOU can do to help. A guide for youth", that also contained a list of local and other resources for screening.	Elaboration Likelihood Model, Theory of Planned Behavior	No intervention	Educational intervention by a relative (untrained)
Nanda et al. 2022	Smartphone-based interventions (apps / SMS / social media campaigns..), Navigation, Reminder	Open-ended text-based scheduling opportunity: SMS reminder + automated scheduling opportunity.	NA	Standard care	NA
Nanda et al. 2020	Telephonic intervention, Navigation	The two-part intervention included (1) phone call and (2) assistance scheduling mammogram. Either a breast nurse navigator, medical assistant, or a mammography technologist called each patient in the intervention group and informed them that they were due for a mammogram. Up to three total attempts were made to contact patients; voicemail was left for patients that did not answer, with patient’s overdue status and a call-back number. All successfully contacted patients were offered the opportunity to schedule a mammogram in real time during the phone call or were left a voicemail with a number (unique to the study) that they could call to schedule their mammogram.	NA	Standard care	NA
Nguyen et al. 2009	Educational intervention, Navigation, Reminder	Outreach activities performed by lay health workers (LHWs) included two small group outreach sessions (90 minutes) for 3-10 women At the first session, LHWs gave a 15- to 20-minute presentation about breast cancer, CBE, and mammography and then led a question-and-answer session. Within 1–2 months, the LHWs contacted participants to explain how to access screening and help with scheduling appointments. The second session occurred ~2 months later, when the LHW answered participants' questions and re-emphasized the benefits of screening. One month later, LHWs called participants to follow up and remind them about the post-intervention survey.	NA	Media campaign	Six Vietmese-language TV and radio advertisements, 13 newspaper advertisements, and six newspaper articles. The campaign also publicized a navigator service and the availability of screening through the federally funded Breast and Cervical Cancer Control Program. Each TV advertisement was shown on two channels with 34 spots monthly; each radio advertisement was broadcast on four stations with 112 spots monthly. Newspaper advertisements were printed in six newspapers or magazines with 72 printed ads monthly. The campaign created and distributed 45,000 bilingual breast cancer–screening booklets, 8500 silk roses with screening reminder cards, and 7500 reminder calendars. Research staff and community representatives (who were not LHWs) distributed these items directly to community

					members at flea markets, cultural events, and community forums, or to staff at physicians' offices, CBOs, temples, pagodas, and churches for distribution over time.	
Pérez-Lacasta et al. 2019	Decision aid	InforMa, a leaflet with detailed information on the benefits and harms of screening	NA	Standard care	Standard leaflet that did not mention harms and recommended accepting the invitation to participate	
Ramirez et al. 2022 (A and V-A)	Smartphone-based intervention (apps / SMS / social media campaigns..)	SMS (Least intervention): Up to 3 text message reminders about the need for a mammogram. Each text message contained a link to a "microsite" (simple website formatted for cell phones) with information about the importance of breast cancer screening and a link to schedule a mammogram online	NA	No intervention	Before	
Ramirez et al. 2022 (B and V-B)	Smartphone-based intervention (apps / SMS / social media campaigns..), Navigation	SMS + navigation (More intervention): Like Least intervention + slightly more educational content on breast health as well as an opportunity to request support from a digital health worker (DHW) to answer questions and assist with mammogram scheduling.	NA	No intervention	Before	
Ramirez et al. 2022 (C and V-C)	Smartphone-based intervention (apps / SMS / social media campaigns..), Navigation, Educational intervention	SMS + navigation + In-person even (Most intervention): Like More intervention + invitation to a "Health Fair" where breast health trained hospital volunteers provided breast cancer risk reduction education, cancer IQ screening, wellness screens, SDOH screening, and assistance scheduling or obtaining mammography. The fairs were held on days with availability for walk-in mammography screenings, and so, attendees could get a mammogram that day if desired. If transportation or childcare was needed, volunteers offered assistance during the pre-visit outreach.	NA	No intervention	Before	
Roberto et al. 2020	Decision aid	Online decision aid, 19 screens, each covering one topic, not printable. Key contents: What is mammography screening? The pros and cons of mammography screening. What might happen in the next 30 years? At what age is mammography screening recommended? The risks related to radiation. Organized mammography screening program, a quality program? What result will the mammography give? What happens at each screening? Diagnostic programs in uncertain cases. Breast density. What is breast cancer and how can it be treated? Differences between false positives and overdiagnosis. The balance between benefits and harms. How are the rates of specific mortality reduction and overdiagnosis measured? Different estimates of the reduction of mortality due to breast cancer. Different overdiagnosis estimates. The decision aid also included an interactive personal page with aspects leading the choice to participate in mammography screening such as values, experience, and perception of the risk of developing BC.	International Patient Decision Aid Standards Collaboration. Nudging-like approach.	Brochure	Web-based standard static brochure	
Savicka et al. 2020	Telephonic intervention, Smartphone-based intervention (apps / SMS / social media campaigns..)	6 GP practices prepared a database of women to be invited with phone numbers; information about the pilot project was posted on the clinic's website and in social networks; information on the possibility to perform oncology screening examinations in the midwife's office was placed at each of the 6 GP offices, as well as at the clinic reception desk, in the branches of the clinic; training of call center employees was conducted on how to address women in the most effective way to use free oncology screening examinations. Women were called. Those who did not pick up the phone also automatically received a text message about the opportunity to visit the midwife's office and perform free examinations (29% of women who did not pick up the phone did not answer unknown callers, but called back having received the text message). Repeated individual interviews were conducted with each of the GP, nurses, encouraging to address women to perform cervical screening and mammography examinations; information about the project was re-posted in social networks and on the clinic's website.	Nudge approach	Before	NA	

Schapira et al. 2019	Decision aid	The decision aid (BCS-PtDA) included the following components: 1) ascertainment of breast cancer risk factors needed for the National Cancer Institute Breast Cancer Risk Assessment Tool (NCI-BCRAT); 2) an introduction to the decision problem (including a description of differing guidelines, overview of risks and benefits including a brief description of overdiagnosis, and the goals of the decision aid); 3) a table outlining United States Preventive Services Task Force and American Cancer Society guidelines; 4) comparison of mortality reduction attributed to mammography between women 40–49 and 50–59 of age; 5) pictographs depicting outcomes of mammography including cancer detection, interval cancers, false positive tests, and true negative tests shown for women aged 40 to 49 and compared with women aged 50 to 59 years of age; 6) pictographs comparing 10-year and lifetime risk for the individual woman to an average-risk woman the same age and 10-year risk compared with an average-risk 50-year-old woman based on the NCI-BCRAT; 7) exemplars that demonstrated women considering the impact of breast cancer risk on their decisions about when to initiate mammography; and 8) an interactive summary sheet where women state their intentions and complete a value clarification exercise where they list the factors most important to them in deciding when to have their first mammogram. The BCS-PDA met 6 out of 6 qualifying criteria for a decision aid, as described in a Delphi consensus survey conducted by the International Patient Decision Aid Standards group. It be viewed at https://www.decide2screen.org/breast/study.aspx	Theoretical framework of SDM and Exemplification theory	Standard care	NA
Sinicrope et al. 2020	Educational intervention, Linguistically adapted intervention	Two 1-2 home visits with a scripted intervention manual and educational materials. Materials developed for this study included: (1) both written and oral English/Navajo versions of the “Glossary for Basic Cancer Terminology in the Navajo Language,” (2) English/Navajo BC prevention brochures from American Cancer Society (written and audio), and (3) family-based digital stories about the cancer experience told by Navajo people.	NA	Standard care	NA
Slater et al. 2017	Printed materials (card, brochure, leaflet, flyer..), Economic support	$25 incentive on completion of a mammogram within 1 year + Folded card with a very brief message about the importance of getting a mammogram and a prompt to “Call your doctor to schedule a mammogram today.” The cards also provided a toll-free number that would connect to Sage, Minnesota’s federally funded breast and cervical cancer screening program for low-income women offering navigation services	Transtheoretical model	Mailed card	Folded card with a very brief message about the importance of getting a mammogram and a prompt to “Call your doctor to schedule a mammogram today.” The cards also provided a toll-free number that would connect to Sage, Minnesota’s federally funded breast and cervical cancer screening program for low-income women offering navigation services
Taymoori et al. 2016 (A)	Educational intervention	8 sessions	Health belief model	Pamphlets	Information according to the American Cancer Society
Taymoori et al. 2017 (B)	Educational intervention	8 sessions	Theory of planned behaviour	Standard care	NA
Tuzcu et al. 2016	Digital-based educational intervention (web, DVD, other platforms..), Invitation letter	Training and consultancy including: 1) a PowerPoint presentation (breast atomy; incidence, mortality, and risk factors of BC; changes in the breast; BSE, CBE, and mammography; the importance of screening; perceived susceptibility, confidence and barriers; benefits of BSE and mammography. 2) A film titled ‘‘How to Do Your Examination’’ with an English version from Bristol-Myers Squibb Oncology (and Turkish translation). 3) BSE training, including observing a study team member performing a BSE, completing silicon breast model exercises, and receiving feedback.4) er cards about BSE and breast cancer screening methods + an invitation cards with a written date and hour for a free mammography (for those aged over 50)	Health Belief Model and Health Promotion Model	Standard care	NA
Wyatt et al. 2022	Educational intervention, Navigation, Culturally sensitive intervention,	Education and media health material and navigation: Educational seminar at mosques, community centers, homes of community members or in parks, with a physical activity component, such as an exercise session, as an additional incentive for women to join. The seminar was provided in-language (i.e., English, Bengali, and Arabic) or with a hired individual	Social marketing theory	Before	NA

	Linguistically adapted intervention	to translate. During the session, women were provided with health education materials (e.g., brochures and palm cards) guided by social marketing theory, developed in several languages (English, Bengali, Arabic, and French), and culturally adapted for Muslim women. Some important components were: guidance on their rights to request a female healthcare provider or a provider who spoke their native language, and information on accessing low-cost or free cancer screenings, including locations and contact information of clinical sites offering screening services. In addition, the materials framed screening as a strategy to maintain a healthy mother role, which aligns with the community's family-centered cultural and gender norms. Filly, women received in-person or phone interactions to provide support on locating and making appointments for screening tests, which also included assistance with transportation.			
